# Integration of microbiome and Koch’s postulates to reveal multiple bacterial pathogens of whitish muscle syndrome in mud crab, *Scylla paramamosain*

**DOI:** 10.1186/s40168-023-01570-6

**Published:** 2023-07-20

**Authors:** Dongwei Hou, Taixin Lian, Guangyu Guo, Han Gong, Chengcheng Wu, Peiyun Han, Shaoping Weng, Jianguo He

**Affiliations:** 1 State Key Laboratory of Biocontrol/School of Marine Sciences, Sun Yat-Sen University, Guangzhou, People’s Republic of China; 2 School of Life Sciences/China-ASEAN Belt and Road Joint Laboratory On Mariculture Technology, Sun Yat-Sen University, Guangzhou, People’s Republic of China; 3 Southern Marine Sciences and Engineering Guangdong Laboratory (Zhuhai), Zhuhai, People’s Republic of China

**Keywords:** Multiple pathogens-one disease, Microbiome, Koch’s postulates, Causative agent, Whitish muscle syndrome, Mud crab, *Scylla paramamosain*

## Abstract

**Background:**

For more than a century, the Koch’s postulates have been the golden rule for determining the causative agents in diseases. However, in cases of multiple pathogens-one disease, in which different pathogens can cause the same disease, the selection of microorganisms that regress infection is hard when Koch’s postulates are applied. Microbiome approaches can obtain relatively complete information about disease-related microorganisms and can guide the selection of target microorganisms for regression infection. In the present study, whitish muscle syndrome (WMS) of *Scylla paramamosain*, which has typical symptoms with whitish muscle and blackened hemolymph was used as an example to establish a new research strategy that integrates microbiome approaches and Koch’s postulates to determinate causative agents of multiple pathogens-one disease.

**Results:**

Microbiome results revealed that *Aeromonas*, *Acinetobacter*, *Shewanella*, *Chryseomicrobium*, *Exiguobacterium*, *Vibrio* and *Flavobacterium*, and *Kurtzmaniella* in hemolymph were bacterial and fungal indicators for WMS. A total of 23 bacteria and 14 fungi were isolated from hemolymph and muscle tissues, and among the bacteria, *Shewanella chilikensis*, *S*. *xiamenensis*, *Vibrio alginolyticus*, *S*. *putrefaciens*, *V*. *fluvialis*, and *V*. *parahaemolyticus* were present in hemolymph and/or muscle tissues in each WMS crab, and the last three species were also present in three Healthy crabs. The target bacteria and fungi were further screened to regression infections based on two criteria: whether they belonged to the indicator genera for WMS, whether they were isolated from both hemolymph and muscle tissues in most WMS crabs. Only *S*. *chilikensis*, *S*. *putrefaciens*, *S*. *xiamenensis*, *V*. *alginolyticus*, *V*. *fluvialis*, and *V*. *parahaemolyticus* met both two criteria. The six bacteria that met both two criteria and six fungi and another bacterium that unmatched any of two criteria were used to perform regression infection experiments based on Koch’s postulates. *S*. *chilikensis*, *S*. *putrefaciens*, *S*. *xiamenensis*, *V*. *alginolyticus*, *V*. *fluvialis*, and *V*. *parahaemolyticus* met both two criteria, and the results indicate that they cause WMS in crabs independently.

**Conclusions:**

This study fully demonstrated that our research strategy that integrates the microbiome and Koch’s postulates can maximize the ability to catch pathogens in one net for the situation of multiple pathogens-one disease.

Video Abstract

**Supplementary Information:**

The online version contains supplementary material available at 10.1186/s40168-023-01570-6.

## Background

Unearthing the pathogeny is a matter of paramount importance in the study of disease. Koch’s postulates [1] have always been the golden rule for determining causative agents for diseases. These postulates include four principals: (i) the microorganism must be present in all diseased individuals, (ii) the microorganism must be isolated from diseased host and be grown in a pure culture, (iii) the re-inoculation of a naive host with this pure culture must lead to the same disease as in original host, and (iv) and the microorganism must be recovered from newly diseased host [2]. For more than a century, these four principals have guided the etiological research. However, different pathogens may cause the same disease in a mechanism called multiple pathogens-one disease. Some viruses, bacteria, and parasites cause diarrhea in children, and their pathogenic mechanisms are not the same [3, 4]. The *Vibrio parahaemolyticus* and *V.* cambei cause acute hepatopancreatic necrosis disease (AHPND) in shrimp with they all carry the virulence genes with PirA and PirB [5]. Based on the concept of multiple pathogens-one disease, the selection of microorganisms that regress infection is difficult under Koch’s postulates.

Recently, the advances in microbiome approaches and microbial culture technologies, and the developments of bioinformatics have driven the field of disease research forward [6–8], thus providing solutions to the abovementioned problems. Microbiome approaches can obtain relatively complete microbial profiles. Hence, it is widely used for determining the relationship between the microorganisms and disease occurrences in many studies. These studies reveal that the significant changes of host-associated microbiota and the abundances of some microbial indicator are closely related to the occurrence of diseases in human [9–11], animal [12–14], and plant hosts [15, 16]. However, these studies only focus on the correlation between indicator taxa and diseases and lack the causality with between these microorganisms and disease occurrence. It is not rigorous to just analyze correlations without culturing disease-related microorganisms to establish this causality [17]. Thus, Koch’s postulates need to be used as reference to verify this cause-and-effect relationship. Considering that microbiome can be effective in identifying disease-related microorganisms, the research strategy that integrate it with Koch’s postulates are likely to be highly effective in unearthing causative agents of diseases [8].

**Fig. 1:**
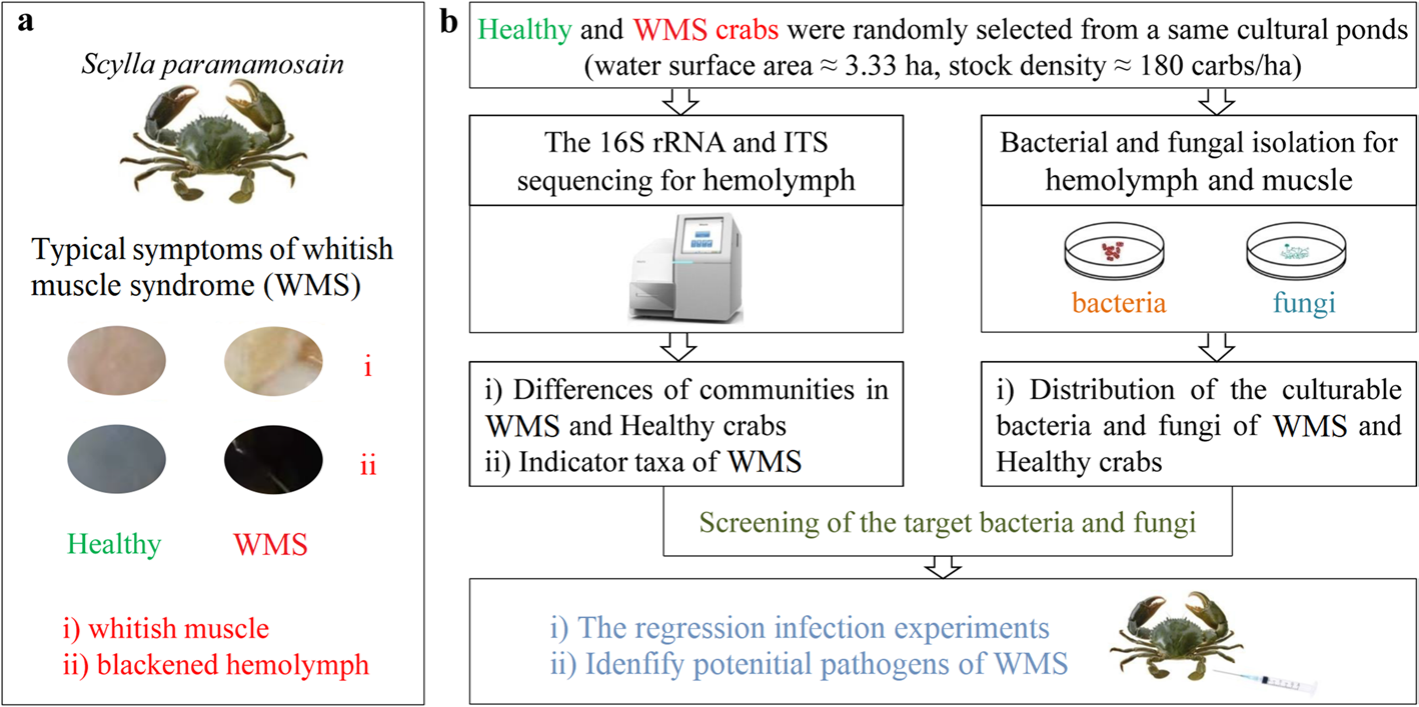
Research strategy that integrates microbiome approaches and Koch’s postulates to determine the causative agents of whitish muscle syndrome (WMS) in *Scylla paramamosain*. **a** Two typical symptoms of WMS with whitish muscle and blackened hemolymph. **b** Overview of our research strategy

The mud crab, *Scylla paramamosain*, is one of the most economically valuable aquatic animals along the coasts of East and Southeast Asia. With the increasing market demand, its cultural industry has rapidly developed in recent years, but the frequent disease outbreaks have severely restricted its development. Especially, the frequent occurrence of whitish muscle syndrome (WMS) has increased harm to *S*. *paramamosain* aquaculture [18]. The typical symptoms of WMS include whitish muscle and blackened hemolymph (Fig. 1a), and host death occurs 4–5 days after onset. The farmers initially linked the occurrence of WMS to the rapid changes of temperature and salinity, because WMS occurs most often when the temperature varies and rain is plentiful [19]. Based on this understanding, the farmers have implemented a series of measures (e.g., adding immune enhancement, using disinfectants, and regulating water quality) to prevent and control WMS in aquaculture, but the effect of these measures is not ideal [20]. *Vibrio*, *Microsporidia*, an unknown virus, and others exist in the muscle tissue of *S*. *paramamosain*, *S*. *serrata*, and *Portunus trituberculatus* with whitish muscle symptom, indicating that this symptom may be related to microorganisms [21]. Moreover, a large number of bacteria are observed, but no parasites are presented in the muscle tissue of *S*. *paramamosain* with WMS (Additional file 2: Figure S1). Therefore, the bacteria (not to exclusion of other microorganisms such as fungi) are most likely involved in the WMS of *S*. *paramamosain*, but none of these studies have determined its causative agents for WMS.

In the present study, the microbiome approaches and Koch’s postulates were integrated, and this new research strategy was implemented to determine the causative agents of WMS in *S*. *paramamosain* (Fig. 1b). The results show that *Shewanella chilikensis*, *S*. *putrefaciens*, *S*. *xiamenensis*, *Vibrio alginolyticus*, *V*. *parahaemolyticus*, and *V*. *fluvialis* all cause WMS independently. This study demonstrates that the strategy that integrates microbiome approaches and Koch’s postulates is feasible and efficient for identifying causative agents for multiple pathogens-one disease.

## Results

### Microbial community diversity in hemolymph of WMS crabs

**Fig. 2:**
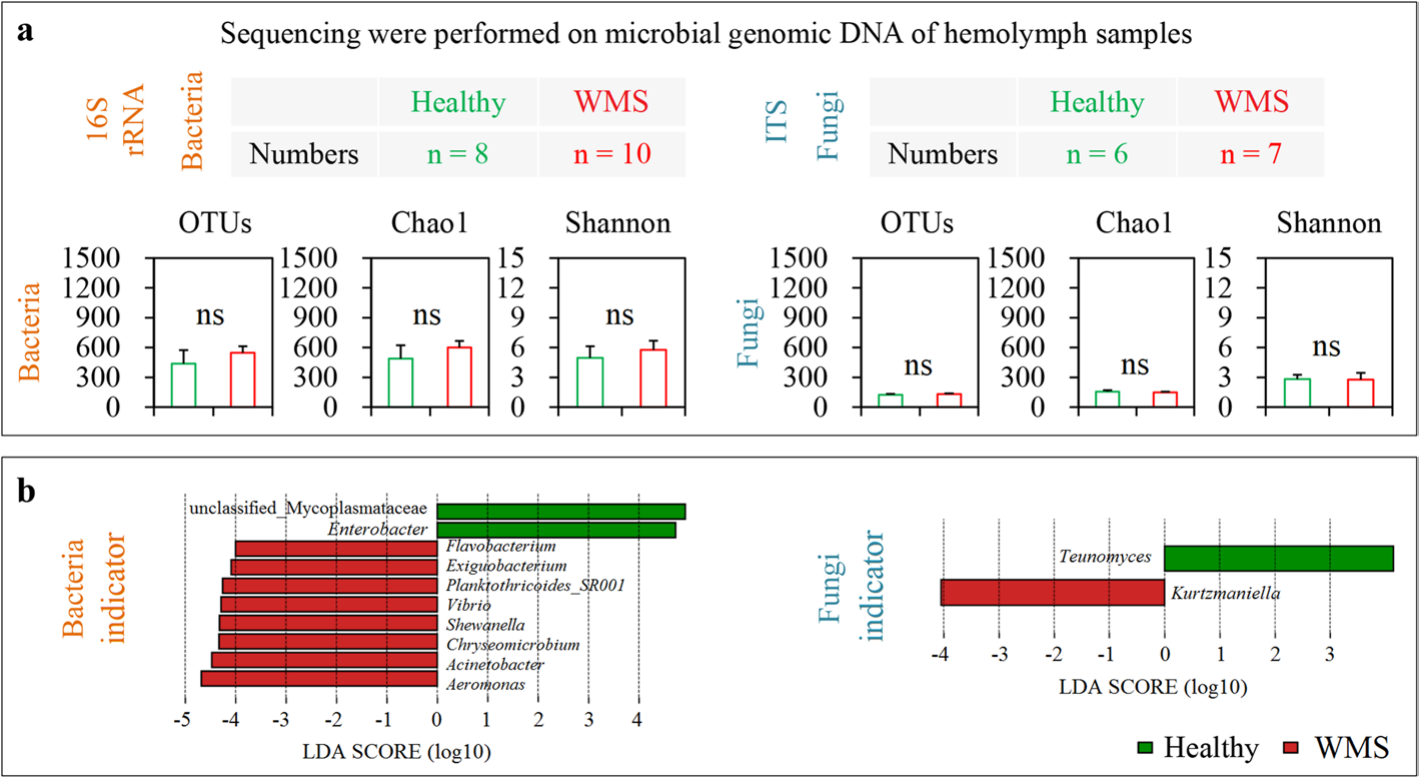
Diversity of bacterial and fungal community in the hemolymph of WMS crabs. **a** Statistical significance of the α-diversity indices of bacterial and fungal community in the hemolymph between WMS and Healthy crabs. **b** LEfSe results of bacterial and fungal indicator genera of WMS. ns: nonsignificance

A total of 74,604 and 71,596 high-quality bacterial and fungal sequences were obtained per sample, and a total of 1,342,872 and 930,748 sequences were retained, respectively (Additional file 2: Figure S2a). Then, the α-diversity indices were calculated using rarefaction curves at the operational taxonomic unit (OTU) level with 1000 iterations, which were stable (Additional file 2: Figure S2a). The bacterial and fungal diversity was compared in hemolymph between WMS and Healthy crabs using 16S rRNA and ITS sequencing, respectively. The bacterial OTU numbers in the hemolymph of WMS crabs (548 ± 65) were higher than that of healthy crabs (438 ± 135), as well as the Chao1 (600 ± 66 and 488 ± 134) and Shannon (5.76 ± 0.92 and 4.97 ± 1.15) indices (Fig. 2a). Fungal OTU numbers (131 ± 9 and 126 ± 9) and the Chao1 (148 ± 8 and 156 ± 15) and Shannon indices (2.79 ± 0.66 and 2.83 ± 0.45) were very nearly the same as those in the hemolymph of WMS and healthy crabs (Fig. 2a). Based on Welch’s *t* test, no significant (*P* > 0.05 in all cases) difference was observed in OTU numbers, and the Chao1 and Shannon indices of bacterial community between WMS and healthy crabs, as well as fungal community (Fig. 2a). The non-metric multidimensional scaling (NMDS) and analysis of similarity (ANOSIM) results showed marked variation in bacterial community structures (ANOSIM *r* = 0.763, *P* = 0.001) in WMS and healthy crabs, but not in fungal community structures (*r* = 0.176, *P* = 0.056) (Additional file 2: Figure S2b). These results revealed that the bacterial community diversity in the hemolymph of WMS and healthy crabs were marked different, but this was not observed in fungi.

### Microbial indicator taxa in the hemolymph of WMS crabs

The most common ten bacterial genera in the hemolymph of WMS crabs were *Aeromonas*, *Enterobacter*, *Acinetobacter*, unclassified_Mycoplasmataceae, *Shewanella*, *Chryseomicrobium*, *Exiguobacterium*, *Vibrio*, Planktothricoides_SR001, and *Pseudomonas*, with average relative abundances of 11.48%, 9.75%, 8.60%, 6.69%, 5.51%, 5.35%, 4.29%, 4.26%, 3.07%, and 2.81%, while those in healthy crabs were unclassified_Mycoplasmataceae, *Enterobacter*, unclassified_Neisseriaceae, unclassified_Sphingomonadaceae, *Acinetobacter*, *Psychrobacter*, *Exiguobacterium*, *Staphylococcus*, unclassified_Rhodobacteraceae, and unclassified_Chitinophagales with abundances of 22.21%, 21.69%, 4.00%, 2.20%, 2.10%, 1.98%, 1.73%, 1.71%, 1.61%, and 1.46%, respectively (Additional file 1: Table S1). The dominant fungal genera of WMS and healthy crabs were *Vanrija*, *Aspergillus*, *Kurtzmaniell*a, unclassified_Ascomycota, *Candida*, *Clavispora*, *Fusarium*, *Wickerhamomyces*, and *Teunomyces*, and the abundance of the first two genera were more than 70% both in WMS and healthy crabs (Additional file 1: Table S2). Thus, the dominant bacterial genera in the hemolymph of WMS and Healthy crabs remarkably differed, but this phenomenon was not observed in fungi.

LEfSe analysis revealed that *Aeromonas*, *Acinetobacter*, *Shewanella*, *Chryseomicrobium*, *Exiguobacterium*, *Vibrio* and *Flavobacterium*, and *Kurtzmaniella* were the microbial biomarkers in hemolymph of WMS (Fig. 2b). Welch’s *t* test results also showed that the relative abundances of these bacterial and fungal genera in the hemolymph of WMS crabs were significant higher (*P* < 0.05 in all cases) than those of healthy crabs (Additional file 2: Figure S2c). Together, *Aeromonas*, *Acinetobacter*, *Shewanella*, *Chryseomicrobium*, *Exiguobacterium*, *Vibrio* and *Flavobacterium*, and *Kurtzmaniella* of hemolymph could act as the indicators for WMS.

### Distribution of bacteria and fungi in the hemolymph and muscle of WMS crabs

**Fig. 3:**
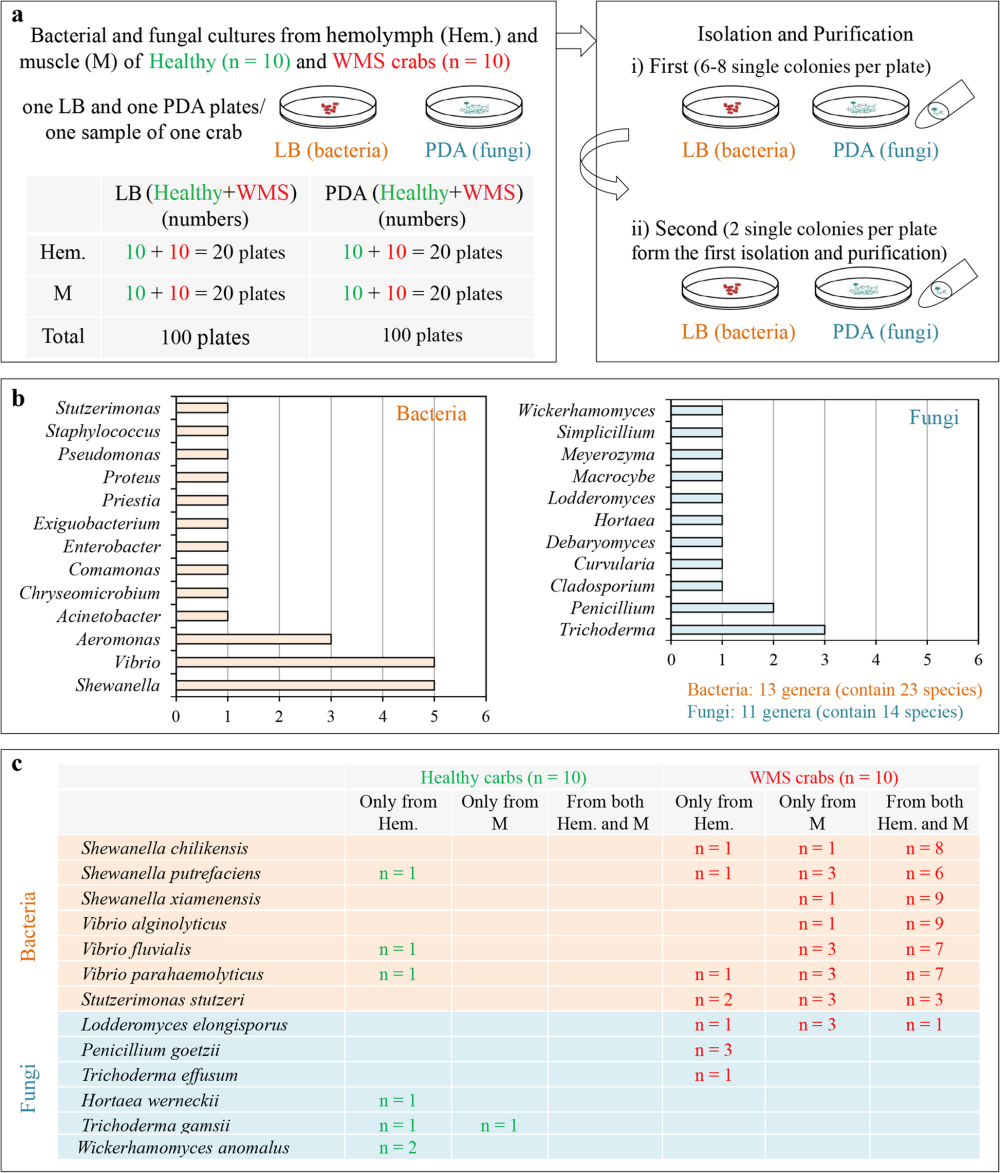
Distribution of culturable bacteria and fungi in hemolymph and muscle tissues of WMS crabs. **a** Process of isolation, purification, and identification of bacteria and fungi. **b** Numbers of genera and species to which all the isolated bacteria and fungi belong. **c** The distribution of sectional bacteria and fungi in hemolymph and muscle of WMS crabs had the preference

A total 23 bacterial and 14 fungal species were isolated from hemolymph and muscle tissues of WMS and healthy crabs (Fig. 3a and b; Additional file 2: Figure S3). In the hemolymph and muscle, 15 and 9 bacterial species were observed in WMS crabs, and 9 and 2 bacterial species were observed in healthy crabs, respectively (Additional file 1: Table S3). While 10 and 3 were observed fungal species in WMS crabs, and 7 and 2 were observed in healthy crabs, respectively (Additional file 1: Table S4). The 23 bacteria were belonged to 13 genera, including *Shewanella* (contained 5 *Shewanella* species), *Vibrio* (5), *Aeromonas* (3), *Acinetobacter* (1), *Chryseomicrobium* (1), *Comamonas* (1), *Enterobacter* (1), *Exiguobacterium* (1), *Priestia* (1), *Proteus* (1), *Pseudomonas* (1), *Staphylococcus* (1), and *Stutzerimonas* (1) (Fig. 3b). The 14 fungi belonged to 11 genera, including *Trichoderma* (3), *Penicillium* (2), *Cladosporium* (1), *Curvularia* (1), *Debaryomyces* (1), *Hortaea* (1), *Lodderomyces* (1), *Macrocybe* (1), *Meyerozyma* (1), *Simplicillium* (1), and *Wickerhamomyces* (1) (Fig. 3b).

Among these 23 bacteria, *S*. *chilikensis*, *S*. *xiamenensis*, *V*. *alginolyticus*, *S*. *putrefaciens*, *V*. *fluvialis*, and *V*. *parahaemolyticus* were present in the hemolymph and/or muscle tissues in each WMS crab (Additional file 1: Table S5). Notably, *S*. *chilikensis* (8 out of 10 WMS crabs), *S*. *putrefaciens* (6), *S*. *xiamenensis* (9), *V*. *alginolyticus* (9), *V*. *fluvialis*. (7), and *V*. *parahaemolyticus* (6) were isolated from both tissues of most WMS crabs (Fig. 3c). *S*. *putrefaciens*, *V*. *fluvialis*, and *V*. *parahaemolyticus* were also isolated from the hemolymph of three different healthy crabs, respectively (Additional file 1: Table S5). In addition, *Stutzerimonas stutzeri* was isolated from both tissues of 3 WMS crabs, and only from the hemolymph and muscle of 2 and 3 WMS crabs, respectively (Fig. 3c). *Acinetobacter johnsonii* (9 and 1) and *Aeromonas bivalvium* (5 and 1) were isolated from the hemolymph of WMS and healthy crabs (Additional file 1: Table S5). By contrast, *S*. *algae* (10 WMS crabs), *Chryseomicrobium imtechense* (9), *V*. *azureus* (7), *Comamonas koreensis* (6), *Proteus penneri* (6), and *S*. *hafniensis* (3) were only isolated from the hemolymph, and *Staphylococcus epidermidis* (7) and *Priestia aryabhattai* (4) isolated from the muscle of WMS crabs (Additional file 1: Table S5). *A*. *hydrophila*, *A*. *media*, *Enterobacter ludwigii*, *Exiguobacterium acetylicum*, *Pseudomonas putida*, and *V. neocaledonicus* were only isolated from a few Healthy crabs (Additional file 1: Table S5).

For fungi, *Lodderomyces elongisporus* (isolated from the hemolymph and muscle of 1 and 3 crabs; and from two tissues of one crab), *Penicillium goetzii* (hemolymph of 3 crabs), and *Trichoderma effusum* (hemolymph of one crab) were only isolated from WMS crabs (Fig. 3c, Additional File 1: Table S6). *Hortaea wernecki*i (hemolymph of one Healthy crab), *Trichoderma gamsii* (hemolymph and muscle of 1 and 1 crab, respectively) and *Wickerhamomyces anomalus* (hemolymph of 2 crabs) were only isolated from Healthy crabs (Fig. 3c, Additional file 1: Table S6). The distribution of the other fungi of WMS crabs had no preference (Additional file 1: Table S5 and S6).

### Multiple *Shewanella* and *Vibrio* species could cause WMS in crabs

**Fig. 4:**
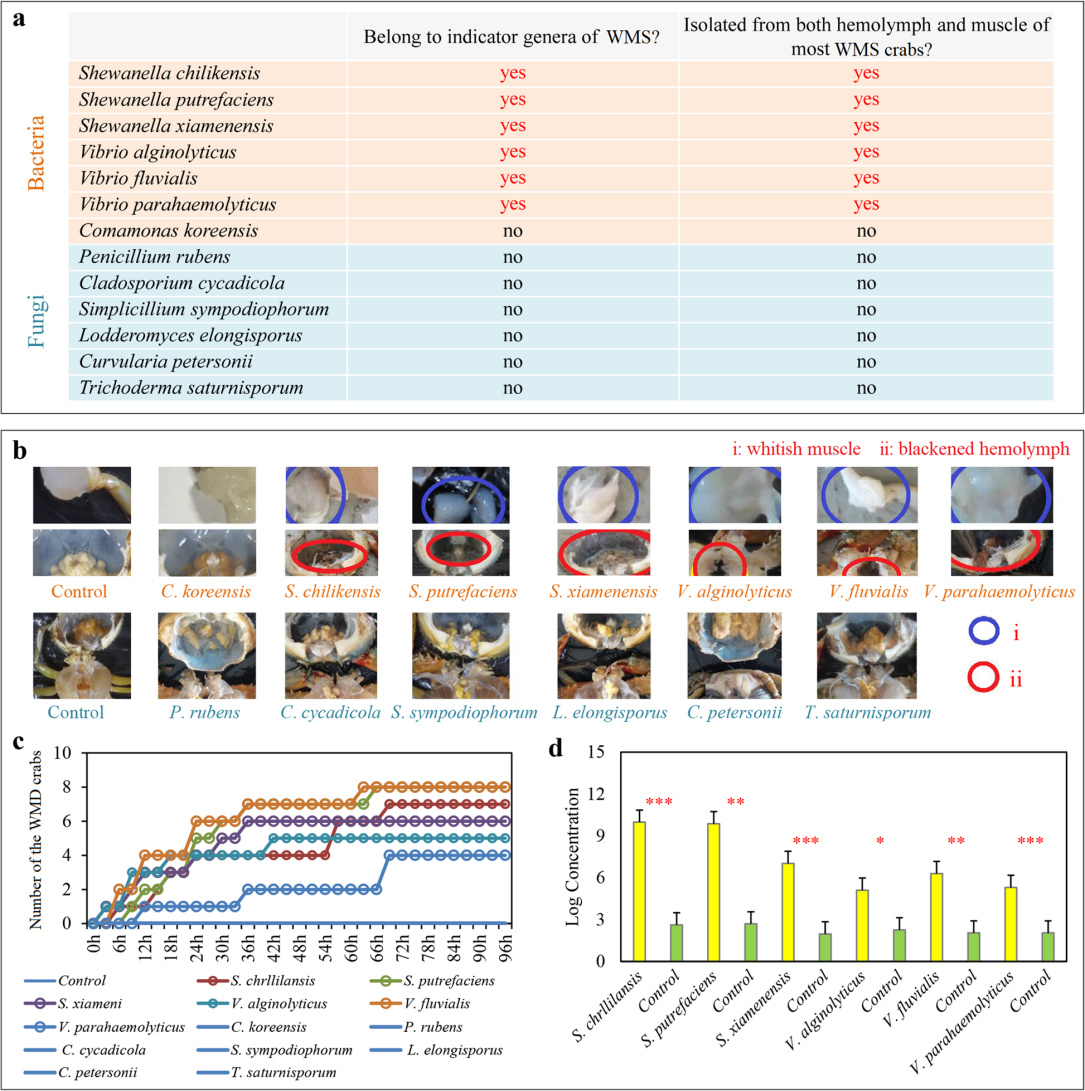
First regression infection experiment based on Koch’s postulates. **a** Two criteria for screening target bacteria and fungi. **b** *S*. *chilikensis*, *S*. *putrefaciens*, *S*. *xiamenensis*, *V*. *alginolyticus*, *V*. *fluvialis*, and *V*. *parahaemolyticus* which met both the two criteria causing WMS with two typical symptoms, but another bacterium and six fungi which unmatched the two criteria did not. **c** Number of WMS crabs in each group at each observation time. **d** Statistical significance of the corresponding bacteria quantity in the hemolymph of WMS crabs in each infection group and that of healthy crabs in the control group. *P < 0.05, **P < 0.01, and ***P < 0.001

The target bacteria and fungi were screened to perform regression infection experiments based on following two criteria: (i) whether they belong to the indicator genera for WMS and (ii) whether they are isolated from both the hemolymph and muscle tissues of most WMS crabs. Only *S*. *chilikensis*, *S*. *putrefaciens*, *S*. *xiamenensis*, *V*. *alginolyticus*, *V*. *fluvialis*, and *V*. *parahaemolyticus* met both criteria. *A*. *johnsonii*, *A*. *bivalvium*, *A*. *hydrophila*, *A*. *media*, and *E*. *acetylicum* matched to the first but not to the second criterion (Additional file 2: Figure S2c; Additional file 1: Table S5), and the remaining bacteria and all fungi did not match either of these two criteria (Additional file 1: Table S5 and S6). Then, these six bacteria that met both two criteria, and another bacterium (*C*. *koreensis*) and six fungi (*Cladosporium cycadicola*, *Curvulari petersonii*, *L*. *elongisporus*, *Penicillium rubens*, *Simplicillium sympodiophorum*, and *Trichoderma saturnisporum*) (Fig. 4a) were selected to perform regression infection experiments twice based on Koch’s postulates.

**Fig. 5:**
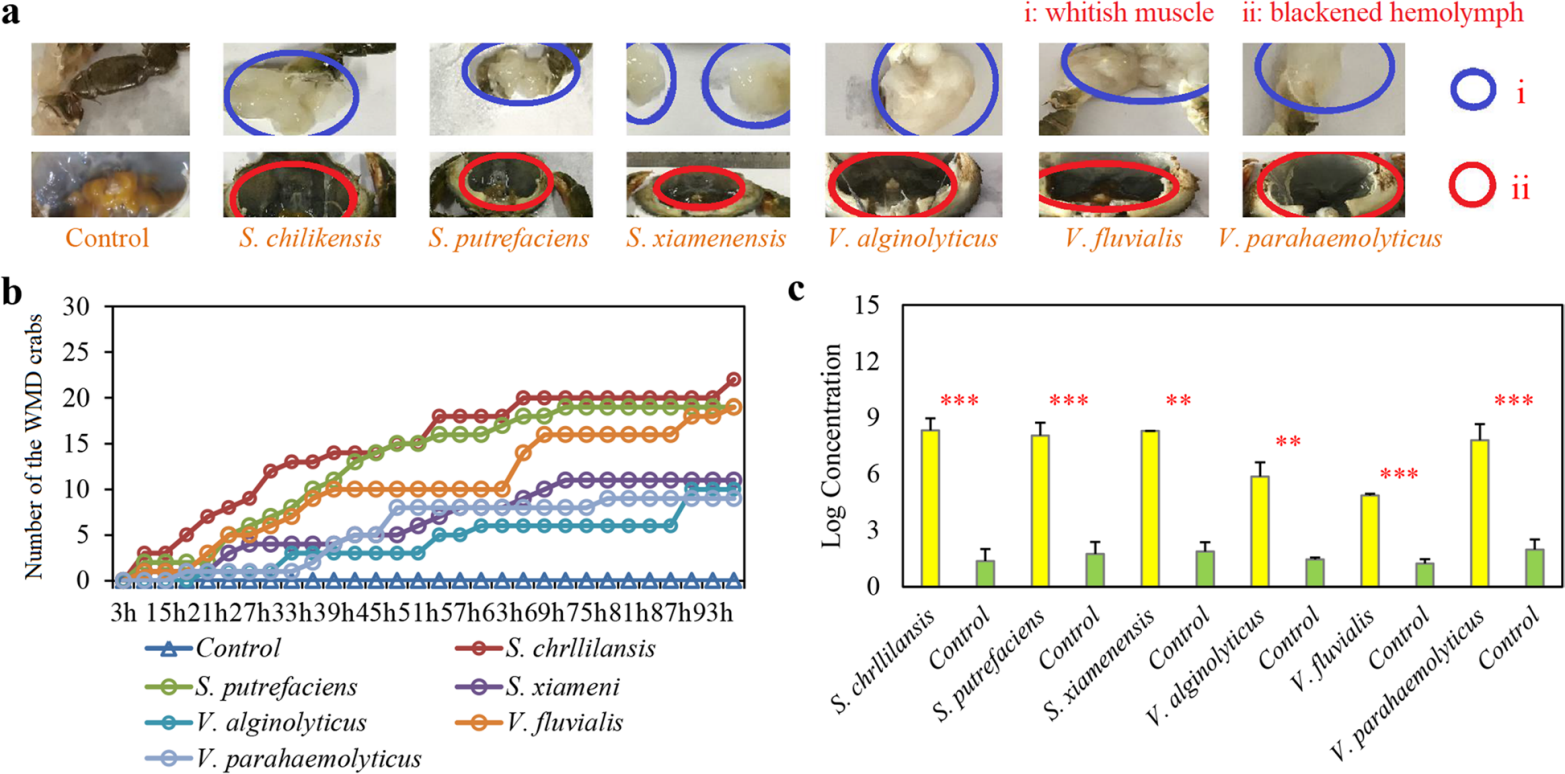
Second regression infection experiment based on Koch’s postulates. **a** *S*. *chilikensis*, *S*. *putrefaciens*, *S*. *xiamenensis*, *V*. *alginolyticus*, *V*. *fluvialis*, and *V*. *parahaemolyticus* caused WMS. **b** Number of WMS crabs in each group at each observation time. **c** Statistical significance of the corresponding bacteria quantity in the hemolymph of WMS crabs in each infection group and that of healthy crabs in the control group. **P < 0.01 and ***P < 0.001

First, these bacteria and fungi were used to infect 10 crabs for each species and found that *S*. *chilikensis* (7 out of 10 crabs), *S*. *putrefaciens* (8), *S*. *xiamenensis* (5), *V*. *alginolyticus* (6), *V*. *fluvialis* (8), and *V*. *parahaemolyticus* (4) could cause crabs to develop whitish muscle and blackened hemolymph, which manifest two typical symptoms of WMS, but another bacterium and all six fungi did not (Fig. 4b and 4c). The RT-qPCR results further showed that the quantity of corresponding bacteria in the hemolymph of WMS crabs in each infection group was 3–7 orders of magnitude higher than that of healthy crabs in the control group (*P* < 0.05 in all cases; Fig. 4d). Then, these six bacteria were used to infect crabs again with three parallels and 12 crabs in each parallel for each species and found that *S*. *chilikensis* (9, 8, 5 out of 12 crabs in each parallel, and total 22 out of 36 crabs), *S*. *putrefaciens* (7, 6, 6, total 19), *S*. *xiamenensis* (5, 5, 3, total 11), *V*. *alginolyticus* (4, 3, 3, total 10), *V*. *fluvialis* (8, 6, 5, total 19), and *V*. *parahaemolyticus* (4, 3, 2, total 11) caused crabs to develop WMS (Fig. 5a and b; Additional file 2: Figure S4). The RT-qPCR results were also consistent with that of the first experiment (Fig. 5c). Moreover, the corresponding bacteria were isolated from the hemolymph of WMS crabs in each group of successful infections in both twice infection experiments (Additional file 2: Figure S5). Therefore, multiple bacteria (*S*. *chilikensis*, *S*. *putrefaciens*, *S*. *xiamenensis*, *V*. *alginolyticus*, *V*. *fluvialis*, and *V*. *parahaemolyticus*) that met our two criteria could cause WMS in* S*. *paramamosain* independently.

## Discussion

For more than a century, Koch’s postulates have been the golden rule for determining causative agents in diseases [1, 2]. The causative agents are difficult to determine instantly and effectively by using Koch’s postulates in cases of multiple pathogens-one disease. In the present study, a new research strategy that integrates microbiome approaches and Koch’s postulates was established, and the results showed that six bacterial species were pathogens for WMS. Our results demonstrated the feasibility and efficiency of this strategy for multiple pathogens-one disease.

Among Koch’s postulates, the key step is the isolation of the pathogen(s) into pure culture from diseased individuals [6]. However, the selection of potential pathogens for regression infection is also troublesome after obtaining pure cultures. Potential pathogens are hard to identify directly form pure culture microorganisms, and many pathogens are also present in healthy individuals, so they cannot be simply judged by their presence or absence in diseased individuals [22–24]. Consequently, some pathogens are not detected instantly and efficiently in some diseases, especially in multiple pathogens-one disease. For instance, *V*. *parahaemolyticus* and *V*. *camberi* are often isolated from the hepatopancreas of shrimp with AHPND, and both species are pathogens of AHPND; however, the discovery dates of these two pathogens have a gap of several years [25–27]. Microbiome approaches can be used to obtain relatively complete disease-related microbial information [9–16] and is considered to can guide the selection of targets for regression infections of Koch’s postulates [8]. Microbiome data were then used to determine the microbial indicators of WMS, which are essential for the subsequent selection of target microorganisms to perform regression infections. Six bacteria (*S*. *chilikensis*, *S*. *putrefaciens*, *S*. *xiamenensis*, *V*. *alginolyticus*, *V*. *parahaemolyticus*, and *V*. *fluvialis*) were determined pathogens for WMS in *S*. *paramamosain*. Thus, the pathogens of WMS should be bacteria, not fungi. The pathogens of WMS may not necessarily be viruses. In WMS crabs, the main virus of is mud crab reovirus, and its abundance is much higher than that of healthy crabs based on viral metagenomics [28]. This virus does not cause WMS in *S*. *paramamosain* [29]. Overall, our research strategy is a wholesale capture to identify the causative agents for the situation of multiple pathogens-one disease.

These six bacterial species which could cause WMS in *S*. *paramamosain* belong to *Shewanella* and *Vibrio*. *Shewanella* and *Vibrio* are disseminated broadly in marine ecosystems, and some of their species are associated with severe diseases for animal and human [30, 31]. *S*. *putrefaciens* and *S*. *xiamenensis* infections are associated with anguillicolosis in *Anguilla Anguilla* [32], while *V*. *alginolyticus* [33], *V*. *parahaemolyticus* [34], and *V*. *fluvialis* [35] cause necrosis, HPNS, and white feces syndrome in *Macrobrachium rosenbergii*, *Litopenaeus vannamei*, and *Penaeus monodon*, respectively. These *Shewanella* and *Vibrio* species are likely to be pathogens for many aquatic animals, and their distribution and dynamics in mariculture need to be focused on. Additionally, the WMS in *S*. *paramamosain* most occurs when the temperature and salinity of water decrease due to a drop in air temperature and a heavy amount of rain. This finding was obtained, because abrupt climate change strongly affects aquaculture ecosystem, and the disturbance of ecosystem affects environment-host–pathogen interactions, resulting in the susceptibility of aquatic animals to bacterial diseases [36, 37]. Farmers and scientists have often observed the outbreaks of vibriosis during the extreme weather in shrimp and crab aquaculture. Notably, frequent extreme weather events caused by the ongoing climate change are likely to further increase the risk of bacterial diseases in cultured animals in aquaculture.

Interestingly, multiple co-infections occurred among six bacterial pathogens in each *S*. *paramamosain* with WMS. Co-infections are very common in nature and occur when the hosts are infected by two or more different pathogens either by simultaneous or secondary infections so that two or more infectious agents are active together in the same host individual [38]. For aquatic animals, this phenomenon is important, but it has not been studied in detail [39]. Notably, co-infections of pathogens result in morbidities and cumulative mortalities in aquatic animals that are often much higher than infected by a pathogen separately [40–42]. In the present study, the six pathogens for WMS are often detected in mariculture ecosystems, leading to increased risk of co-infection for hosts by these pathogens. Thus, the interactions among six pathogens and their pathogenicity and ability to cause WMS deserve further investigation.

Taken together, noteworthy aspects of our study include the synergistic integration of independent- and dependent-culture techniques by using high-throughput and pure culture approaches. Although much effort in the study of Koch’s postulates has been directed towards the identification of causative agents for diseases, our findings fully demonstrate that microbiome approaches need to be integrated for multiple pathogens-one disease. Six bacterial pathogens (*S*. *chilikensis*, *S*. *putrefaciens*, *S*. *xiamenensis*, *V*. *alginolyticus*, *V*. *parahaemolyticus*, and *V*. *fluvialis*) were observed in diseased individuals, and each species causes WMS in *S*. *paramamosain* independently. This study provided a successful case for the research strategy that integrates microbiome approaches and Koch’s postulates to determine the causative agents for multiple pathogens-one disease.

## Materials and methods

### Experimental design and sample collection

The pathogens of WMS in *S*. *paramamosain* were identified by selecting 13 WMS and 10 healthy crabs in the same pond (water surface area, 3.33 ha; stock density, 180 carbs^.^ha^−1^). The samples were obtained from Longxue Island Aquaculture Farm, Nansha, Guangzhou, China, in September 18, 2021). Then, the hemolymph was collected for bacterial and fungal sequencing, and the hemolymph and muscle were obtained for bacterial and fungal culture (sample information was detailed in Additional file 1: Table S7 and S8. Finally, the target microorganisms were screened based on sequencing and culture data, and regression infections were performed to identify pathogens based on Koch’s postulates. In the sampling process, the hemolymph was quickly absorbed and moved into a centrifuge tube containing a 1.0 mL of anticoagulant. Then, a 50-mg muscle was collected about in a 2.0-mL sterile centrifuge tubes containing 500 μL of PBS (1 ×) and 3.5-mm sterile steel balls. Samples were placed in a container with ice and immediately transported to the laboratory. The samples for sequencing were stored at − 80℃ until nucleic acid extraction, and the samples for culture were stored at 4℃ until usage for culture.

### 16S rRNA gene and ITS amplicon sequencing

Hemolymph DNA was extracted using the microbial DNA extraction mini kit (Mabio, Guangzhou, Guangdong, China). The V3-V4 regions of the 16S rRNA gene were amplified by the 338F and 806R, and the ITS1 region was amplified by the ITS1-F and ITS1-R. Their PCR products were combined equally and sequenced using the Illumina MiSeq platform (Illumina, San Diego, USA) at the MAGIGENE Biological Technology Co., Ltd., (Guangzhou, China). FLASH was used to merge the paired-end sequences, and QIIME2 (version 2020.6.0) was employed to process the merged sequences [43, 44]. The chimeric sequences were removed using UCHIME [45]. A distance-based identity of sequences with ≥ 97% were grouped into OTUs by using vsearch (version 2.8.1). Finally, the RDP classifier algorithm (Silva SSU database 138) was used to select the most abundant sequence from OTU as a representative.

### Isolation, purification, and identification of bacteria and fungi

The samples for microbial culture were ground using the freeze grinder (50 Hz 10 min at 4℃) and moved to a refrigerated centrifuge (5000 rpm 3 min at 4℃). Approximately, 100 µL of turbid fluid was diluted by 10,000 times, and then, 50µL of sample was placed in the LB and PDA agar plates, which were cultured in incubators for 16 and 24 h at 37℃ and 28℃, respectively [46–48]. The single colony was randomly selected under the characteristics of shape and color and then purified on a new plate. The inoculating loop was burned on an alcohol lamp for 1 min, and when cooling, the single colony was selected and spread with a continuous line on a new plate. It was then placed in an incubator for cultivation, and each colony was purified twice or thrice. Then, 8–10 colonies from each last streak plate were selected, and each colony was inoculated into a sterile centrifuge tube with 1.0 mL of liquid medium for expanding culture [49, 50]. The mold mycelium was picked on new PDA plates which were cultured in an incubator about 3–5 days at 28℃, and then, each colony was inoculated into a slant culture-medium for expanding culture [46]. Then, the purified bacteria and fungi were directly amplified by PCR, and yeast spores were subjected to nucleic acid extraction (FastPure®Cell/Tissue DNA Isolation Mini Kit, Vazyme Biotech Co. Ltd.) before PCR (the primers: 27-F and 1429-R for bacteria and ITS1-F and ITS4-R for fungi) [51–53]. The specificity and integrity of PCR products were examined by gel electrophoresis. The PCR products were further sequenced by Tianyi Huiyuan Biotechnology Co. Ltd., and the sequences were compared to species data in the NCBI BLAST website (https://blast.ncbi.nlm.nih.gov/Blast.cgi). Sequences with homology similarity greater than 98.5% were selected, and the BLAST results were downloaded as FASTA files. Then, these and our sequence files were imported into MEGA6; then, the phylogenetic tree was constructed using the maximum likelihood method to determine their species or related species and then annotated to genus and species level [54]. All Sanger sequence information is summarized in Additional file 3.

### Regression infection experiments of bacteria and fungi to crabs

Based on sequencing and culture data, the target bacteria and fungi were screened, and twice regression infection experiments were performed based on the following Koch’s postulates. First, the targets were infected with 10 crabs for each species. In addition, the bacteria that caused WMS in the first experiment were infected with crabs again, and this process involved three parallels and 12 crabs in each parallel for each group. The crabs used in twice challenge experiments were obtained from different farms in Zhuhai city at different culturing batches, and twice experiments were conducted in Lianxi Experimental Base of Chinese Academy of Fishery Sciences, Doumen, Zhuhai, from December 2021 to January 2022 and April 2011. All crabs were free of WMS and other diseases, and their body length and weight were 9.0 ± 0.6 cm and 450.0 ± 45.0 g, respectively. The water in each aquarium had a volume of 0.5 m^3^, depth of 25.0 cm depth, and salinity of 3‰ at indoor temperature [55]. Before infections, the bacterial OD values were calculated, and the concentration standard curve of each strain was constructed. The specific steps were as follows: (i) the bacteria solution was diluted for 10^4^, 10^5^, 10^6^, and 10^7^ times. Approximately, 100 µL of diluent was absorbed and coated in new LB/PDA plates with coating sticks for three times and then incubated overnight in incubators at 37℃ (the optimal counting multiple which with 80–200 colonies; optimal dilution ratio, 10^6^ time); (ii) the solution was diluted by 2, 4, 6, 8, and 10 times, and OD values were determined using NanoVuePlus spectrophotomete (GE Healthcare, Boston, USA). Each gradient was determined for three times, and the average value was calculated. These bacterial solutions were further diluted for 10^6^, and 100 µL of diluent was absorbed and coated in new plates with coating sticks for three times. The samples were then incubated overnight at 37℃ incubators, and the colonies were counted; (iii) the regression curve of average OD values and colony numbers was generated to calculate subsequent concentration, in which the abscissa represents the OD, and the ordinate represents the colony numbers (× 10^6^) [56, 57]. The calculation method of yeast concentration was consistent with that of bacteria. The spores of molds are needed for regression infections. In this process, the molds were cultured with PDA slant culture-medium at 25℃ for 7 days, and the spore suspension was obtained by scraping or washing surface spores with 5 mL of sterile water containing 50 µL of 0.1% Tween-80 solution [58]. The spore suspension was placed in a sterilized 50-mL triangular flask with sterile glass spheres, thoroughly shaken and filtered through a filter membrane. Then, the filtrate residue was rinsed for 2–3 times with sterile water until the filtrate volume reached 100 mL. Lastly, the spore suspension was diluted for 1000 times with sterile water, and the clean blood counting plates were taken for counting.

When counting, the cover glasses were placed on counting area, 20 µL of diluent was absorbed, and a small drop was dropped along the lower edge of cover glass from groove on both sides of middle platform of counting plate. The cover glass was added and allowed to stand for 5 min. After counting, the chamber was found at low magnification and converted to high magnification for observation and counting. The spore number *s* in the upper left, lower left, upper right, and lower right grids was calculated according to the diagonal direction with 16 grids, namely 100 grids. Each sample was subjected to this process for two to three times to calculate the spore numbers. Each spore suspension was diluted by 10^3^ times and counted for three times in each counting area. The average value was used to calculate corresponding spore number, and the spore suspension was diluted to infection concentrations. The formula for spore calculation in as follows [59]:

$$\mathrm{Spores number }(\mathrm{M})=\frac{\mathrm{number of spores in }80\mathrm{ small grids }}{80}\times 400\times {10}^{4}\times \mathrm{dilution multiple}$$

The bacteria and yeast solution were precipitated at 3000 rpm and at 25℃ for 5 min and then the supernatant was removed. Sediments were blown and suspended with 50 mL of sterilized 0.85% normal saline to make the suspension. Then, the suspension was diluted to 5 × 10^6^ CFU^.^mL^−1^ with 0.85% normal saline based on the OD values and regression curve formulas. Similarly, the suspension for each mold was prepared with 5 × 10^6^ spores in 1.0 mL of suspension containing 10 µL of 0.1% Tween-80 solution. The crabs were infected through injection into the base of swimming feet with 1.0 mL sterile syringes at an angle of 45° towards heart with 100 µL of suspensions for each crab in the infection groups. Approximately, 100 µL of 0.85% saline was administered to each crab in the control group. Moreover, the mold infection required the addition of a control group by injecting 100 µL of 0.85% normal saline containing 10 µL of 0.1% Tween-80 solution. All crabs were not fed for 24 h before infections. After infection, the healthy status of crabs of each group was recorded and photographed every 6 h. The hemolymph was sampled from all WMS crabs and three healthy crabs (only at 96 h). A little hemolymph was used for culture, and the remainder was stored in anticoagulant for RT-qPCR to detect the quantity of corresponding infection species [60]. The detailed primers are shown in Additional file 1: Table S9. Also, the Sanger sequence information is shown in Additional file 3.

### Statistical analysis

Welch’s *t* test was employed to compare the bacterial/fungal α-diversity indices in hemolymph between WMS and healthy crabs. LEfSe and Welch’s *t* test were used to determine the bacterial and fungal indicators in hemolymph of WMS at the genus level (because the V3-V4 regions and ITS1 region with short reading length did not have very accurate identification ability for bacteria and fungi at the species level, in contrast, the result is accurate at the genus level). NMDS and ANOSIM were used to evaluate the community structure differences of these two groups based on Bray Curtis distance. Welch’s *t* test was used to identify the differences in the quantity of bacteria which caused WMS between WMS crabs of each infection group and healthy crabs of the control group based on twice regression infection experiments.

## Supplementary Information

**Additional file 1: Table S1.** The relative abundance of bacterial taxa in the hemolymph of WMS and Healthy crabs at the genus level. **Table S2.** The relative abundance of fungal taxa in the hemolymph of WMS and Healthy crabs at the genus level. **Table S3.** Sequence, taxonomy, and other information of isolated bacterial species. **Table S4.** Sequence, taxonomy, and other information of isolated fungal species. **Table S5.** Distribution of the isolated bacteria in the hemolymph and muscle tissues of each WMS and Healthy crab. **Table S6.** Distribution of the isolated fungi in the hemolymph and muscle tissues of each WMS and Healthy crab. **Table S7.** Sample information for 13 WMS and 10 Healthy selected crabs. **Table S8.** Sample information for the 16S rRNA and ITS amplicon sequencing in the hemolymph of WMS and Healthy crabs. **Table S9.** Primer pairs of RT-qPCR for six bacteria which cause WMS in twice regression infection experiments.

**Additional file 2: Fig. S1.** A large number of bacteria (the red arrow points to the blue dot) but no parasites were present in the muscle of WMS crabs using the brightfield, fluorescence & FISH digital pathology scanner (Lecia, Versa 8). **Fig. S2.** Bacterial and fungal community of WMS. a Rarefaction and Shannon curve. b Bacterial and fungal community structure of WMS and Healthy crabs based on Bray-Curtis distance. c Bacterial and fungal indicator genera in the hemolymph for WMD. *: *P* < 0.05, ***: *P* <0.001. **Fig. S3.** Phylogenetic tree of all 23 bacterial species isolated. **Fig. S4.** Sequence chart of WMS in each infection group during the second regression infection experiment. **Fig. S5.** Phylogenetic tree of *S. chilikensis*, *S. putrefaciens*, *S. xiamenensis*, *V. alginolyticus*, *V. fluvialis*, and *V. parahaemolyticus* re-isolated which cause WMS in twice regression infection experiments.

**Additional file 3.** Bacteria. Second challenge. First challenge. Fungi.

## Data Availability

The high-throughput sequencing data used in this study are available in the NCBI Short Read Archive under Bioproject ID PRJNA905173.

## Abbreviations

AHPND: Acute hepatopancreatic necrosis disease
WMS: Whitish muscle syndrome
OTU: Operational taxonomic unit
NMDS: Non-metric multidimensional scaling
ANOSIM: Analysis of similarity
