# Supplementary material for: Integration of microbiome and Koch’s postulates to reveal multiple bacterial pathogens of whitish muscle syndrome in mud crab, Scylla paramamosain
Source: Microbiome. 2023 Jul 20;11:155. doi: 10.1186/s40168-023-01570-6 (PMC10357871; doi:10.1186/s40168-023-01570-6)
Supplement: Supplementary file 2 — Additional file 1: Table S1. The relative abundance of bacterial taxa in the hemolymph of WMS and Healthy crabs at the genus level. Table S2. The relative abundance of fungal taxa in the hemolymph of WMS and Healthy crabs at the genus level. Table S3. Sequence, taxonomy, and other information of isolated bacterial species. Table S4. Sequence, taxonomy, and other information of isolated fungal species. Table S5. Distribution of the isolated bacteria in the hemolymph and muscle tissues of each WMS and Healthy crab. Table S6. Distribution of the isolated fungi in the hemolymph and muscle tissues of each WMS and Healthy crab. Table S7. Sample information for 13 WMS and 10 Healthy selected crabs. Table S8. Sample information for the 16S rRNA and ITS amplicon sequencing in the hemolymph of WMS and Healthy crabs. Table S9. Primer pairs of RT-qPCR for six bacteria which cause WMS in twice regression infection experiments. [file 40168_2023_1570_MOESM1_ESM.docx]

**Supplementary Tables**

**Supplementary Table 1 The relative abundance of bacterial taxa in the hemolymph of WMS and Healthy crabs at the genus level**

|  | Healthy_hemolymph | | WMS_hemolymph | |
| --- | --- | --- | --- | --- |
| Bacterial Genera | mean | sd | mean | sd |
| *Enterobacter* | 21.69304% | 13.41489% | 9.74733% | 7.53866% |
| unclassified_Mycoplasmataceae | 22.21094% | 16.65675% | 6.69428% | 14.77864% |
| *Aeromonas* | 1.21073% | 0.63842% | 11.48249% | 7.40899% |
| *Acinetobacter* | 2.10126% | 0.93339% | 8.59820% | 4.41701% |
| *Chryseomicrobium* | 0.88283% | 0.60629% | 5.35427% | 5.15884% |
| *Shewanella* | 0.67071% | 0.61145% | 5.51096% | 5.58046% |
| *Exiguobacterium* | 1.72544% | 1.26792% | 4.28851% | 2.36849% |
| unclassified_Neisseriaceae | 4.00079% | 6.16608% | 1.58919% | 2.36918% |
| *Vibrio* | 0.14745% | 0.13492% | 4.25781% | 3.19099% |
| *Pseudomonas* | 1.36990% | 0.92451% | 2.80722% | 1.39898% |
| Planktothricoides_SR001 | 0.00318% | 0.00312% | 3.07101% | 4.29923% |
| *Flavobacterium* | 0.60285% | 0.48486% | 2.57386% | 2.22664% |
| *Psychrobacter* | 1.97627% | 2.10217% | 1.08882% | 0.72965% |
| *Staphylococcus* | 1.71103% | 1.45162% | 0.95960% | 0.72994% |
| *Bacteroides* | 1.42737% | 2.51980% | 1.15195% | 2.08943% |
| unclassified_Sphingomonadaceae | 2.19928% | 1.37090% | 0.49394% | 0.29851% |
| unclassified_Rhodobacteraceae | 1.61151% | 1.00603% | 0.63884% | 0.28581% |
| *Hydrogenophaga* | 0.01206% | 0.00975% | 1.52150% | 0.91427% |
| *Paracoccus* | 1.38230% | 0.75625% | 0.30950% | 0.15343% |
| *Alishewanella* | 0.01056% | 0.00427% | 1.34242% | 0.87260% |
| unclassified_Chitinophagales | 1.45853% | 2.24890% | 0.06421% | 0.07554% |
| unclassified_Enterobacteriaceae | 0.73957% | 0.59411% | 0.62584% | 0.49862% |
| unclassified_Rhizobiaceae | 1.24608% | 0.49281% | 0.12318% | 0.10772% |
| Cyanobium_PCC_6307 | 1.15795% | 1.48574% | 0.09986% | 0.07646% |
| *Escherichia Shigella* | 0.56448% | 0.98542% | 0.51284% | 0.99407% |
| *Lactococcus* | 0.64457% | 0.47645% | 0.43282% | 0.35082% |
| *Candidatus* Hepatoplasma | 1.15828% | 2.45705% | 0.00657% | 0.00951% |
| *Rheinheimera* | 0.01123% | 0.00411% | 0.88655% | 0.70143% |
| *Comamonas* | 0.10740% | 0.08584% | 0.79661% | 0.53672% |
| unclassified_Bacteria | 0.54169% | 0.59696% | 0.44595% | 0.32299% |
| unclassified_Rhodocyclaceae | 0.08796% | 0.07290% | 0.78682% | 0.93512% |
| unclassified_Absconditabacteriales__SR1 | 0.99861% | 0.60789% | 0.01836% | 0.02799% |
| *Sphingomonas* | 0.51556% | 0.36550% | 0.39502% | 0.52305% |
| *Labrenzia* | 0.94197% | 1.61488% | 0.04705% | 0.05779% |
| *Rubritalea* | 0.92204% | 0.68370% | 0.02574% | 0.03681% |
| *Pseudarcobacter* | 0.02832% | 0.02105% | 0.71042% | 0.96622% |
| unclassified_Weeksellaceae | 0.63267% | 0.60060% | 0.21192% | 0.14343% |
| unclassified_Saprospiraceae | 0.64340% | 0.48762% | 0.18364% | 0.12621% |
| unclassified_Flavobacteriaceae | 0.57755% | 1.17405% | 0.21098% | 0.16660% |
| *Streptococcus* | 0.40078% | 0.52671% | 0.34073% | 0.56653% |
| unclassified_Saccharimonadales | 0.79252% | 0.65988% | 0.02654% | 0.02696% |
| *Uruburuella* | 0.47652% | 0.34641% | 0.27157% | 0.22100% |
| *Hypnocyclicus* | 0.07707% | 0.20666% | 0.55212% | 0.98972% |
| uncultured_Candidatus_Saccharibacteria_bacterium | 0.57839% | 0.43205% | 0.06769% | 0.06265% |
| *Weissella* | 0.09333% | 0.08805% | 0.43322% | 0.38697% |
| ZOR0006 | 0.56867% | 0.92931% | 0.04732% | 0.09517% |
| unclassified_Microtrichaceae | 0.60855% | 0.71422% | 0.01541% | 0.02597% |
| *Ralstonia* | 0.24764% | 0.22888% | 0.29529% | 0.24532% |
| *Corynebacterium* | 0.50533% | 0.37846% | 0.07332% | 0.07993% |
| *Delftia* | 0.33963% | 0.23097% | 0.20495% | 0.17017% |
| LD29 | 0.03736% | 0.04398% | 0.44555% | 0.48104% |
| *Chryseobacterium* | 0.36509% | 0.26604% | 0.18243% | 0.11977% |
| unclassified_Comamonadaceae | 0.09651% | 0.10637% | 0.38550% | 0.26204% |
| *Taibaiella* | 0.49880% | 0.71729% | 0.06085% | 0.06658% |
| *Rhodobacter* | 0.30695% | 0.14658% | 0.18806% | 0.10574% |
| unclassified_JGI_0000069_P22 | 0.47769% | 0.34439% | 0.04946% | 0.05099% |
| *Fusibacter* | 0.22385% | 0.36268% | 0.24476% | 0.30180% |
| *Ilumatobacter* | 0.39710% | 0.36644% | 0.09959% | 0.09093% |
| *Thiothrix* | 0.46479% | 0.57436% | 0.04021% | 0.08148% |
| unclassified_Nostocaceae | 0.40162% | 0.59024% | 0.08552% | 0.09628% |
| unclassified_Micrococcaceae | 0.30746% | 0.29827% | 0.15951% | 0.10303% |
| *Fluviicola* | 0.01910% | 0.02994% | 0.37210% | 0.41917% |
| *Domibacillus* | 0.20324% | 0.16533% | 0.21473% | 0.17771% |
| *Stenotrophomonas* | 0.22469% | 0.15545% | 0.18163% | 0.13943% |
| *Lacticaseibacillus* | 0.23776% | 0.18317% | 0.14235% | 0.15702% |
| *Prevotella_9* | 0.21581% | 0.45006% | 0.15616% | 0.35412% |
| *Ruegeria* | 0.36325% | 0.55861% | 0.02037% | 0.03363% |
| unclassified_Aeromonadaceae | 0.00771% | 0.00694% | 0.30441% | 0.28845% |
| *Cloacibacterium* | 0.01290% | 0.01700% | 0.29288% | 0.26676% |
| *Draconibacterium* | 0.27931% | 0.46530% | 0.07573% | 0.11860% |
| unclassified_Gemmatimonadaceae | 0.11628% | 0.20522% | 0.19382% | 0.38420% |
| CL500_3 | 0.01743% | 0.03783% | 0.27237% | 0.22095% |
| *Albimonas* | 0.34113% | 0.39790% | 0.01327% | 0.01810% |
| unclassified_Cyanobacteriia | 0.00050% | 0.00100% | 0.28256% | 0.30344% |
| *Cetobacterium* | 0.24228% | 0.40975% | 0.08136% | 0.09634% |
| *Erythrobacter* | 0.23022% | 0.13784% | 0.08552% | 0.08311% |
| unclassified_Stappiaceae | 0.22234% | 0.20716% | 0.08726% | 0.08675% |
| *Parabacteroides* | 0.14024% | 0.26590% | 0.14785% | 0.31097% |
| *Porphyromonas* | 0.16789% | 0.12461% | 0.11755% | 0.16991% |
| unclassified_Muribaculaceae | 0.14795% | 0.26219% | 0.13337% | 0.31975% |
| *Bacillus* | 0.17325% | 0.18671% | 0.09932% | 0.06074% |
| *Arcobacter* | 0.11745% | 0.11032% | 0.13806% | 0.13232% |
| Allorhizobium_Neorhizobium_Pararhizobium_Rhizobium | 0.15515% | 0.13640% | 0.10750% | 0.15674% |
| *Taeseokella* | 0.23893% | 0.29360% | 0.03324% | 0.03441% |
| *Vogesella* | 0.00184% | 0.00312% | 0.21956% | 0.15081% |
| *Halarcobacter* | 0.00989% | 0.01329% | 0.20187% | 0.40203% |
| *Faecalibacterium* | 0.11879% | 0.21907% | 0.11353% | 0.24497% |
| *Deinococcus* | 0.10824% | 0.07130% | 0.12023% | 0.15947% |
| unclassified_Vicinamibacteraceae | 0.10790% | 0.23134% | 0.11045% | 0.12075% |
| CK06_06_Mud_MAS4B_21 | 0.00050% | 0.00069% | 0.19583% | 0.25018% |
| *Fusobacterium* | 0.18414% | 0.25362% | 0.04316% | 0.07204% |
| *Elizabethkingia* | 0.11544% | 0.08374% | 0.08967% | 0.08339% |
| *Pantoea* | 0.03921% | 0.05325% | 0.14450% | 0.09325% |
| *Pseudoalteromonas* | 0.00620% | 0.00656% | 0.16782% | 0.20499% |
| uncultured_Acidobacterium_sp. | 0.04306% | 0.08068% | 0.13337% | 0.09873% |
| *Mangrovibacterium* | 0.08914% | 0.09542% | 0.09490% | 0.09370% |
| unclassified_Hydrogenophilaceae | 0.00034% | 0.00095% | 0.16460% | 0.18175% |
| Nodosilinea_PCC_7104 | 0.00603% | 0.01600% | 0.15750% | 0.17623% |
| *Kurthia* | 0.01676% | 0.03757% | 0.14865% | 0.16294% |
| RB41 | 0.09986% | 0.17890% | 0.08163% | 0.17306% |
| *Acholeplasma* | 0.00117% | 0.00112% | 0.15777% | 0.25363% |
| uncultured_Bacteroidetes_bacterium | 0.02212% | 0.05070% | 0.14088% | 0.25069% |
| *Bradyrhizobium* | 0.08193% | 0.07138% | 0.08833% | 0.10132% |
| *Roseibacillus* | 0.17425% | 0.11241% | 0.01287% | 0.02042% |
| Prochlorothrix_PCC_9006 | 0.00000% | 0.00000% | 0.15093% | 0.20662% |
| *Dechlorobacter* | 0.00720% | 0.01663% | 0.14275% | 0.16387% |
| Sphaerospermopsis_BCCUSP55 | 0.00000% | 0.00000% | 0.14852% | 0.19017% |
| *Algoriphagus* | 0.01793% | 0.03136% | 0.13310% | 0.11568% |
| unclassified_Candidatus_Kaiserbacteria | 0.00955% | 0.02145% | 0.13967% | 0.09642% |
| *Enterococcus* | 0.09986% | 0.05673% | 0.06689% | 0.05116% |
| *Myroides* | 0.06049% | 0.06980% | 0.09798% | 0.08462% |
| *Brevundimonas* | 0.09266% | 0.07177% | 0.07185% | 0.07460% |
| unclassified_Kapabacteriales | 0.03870% | 0.03048% | 0.11233% | 0.11049% |
| *Lysobacter* | 0.04206% | 0.04718% | 0.10951% | 0.08681% |
| *Anderseniella* | 0.15163% | 0.10450% | 0.02171% | 0.02128% |
| Candidatus_Megaira | 0.01458% | 0.02346% | 0.12747% | 0.08461% |
| *Sphingobacterium* | 0.04306% | 0.05803% | 0.10361% | 0.08053% |
| unclassified_Enterobacterales | 0.03385% | 0.02415% | 0.10763% | 0.13611% |
| *Candidatus* Bacilloplasma | 0.15817% | 0.31820% | 0.00536% | 0.00586% |
| unclassified_Gaiellales | 0.07171% | 0.13419% | 0.07238% | 0.12077% |
| unclassified_Unknown_Family | 0.06585% | 0.14955% | 0.07587% | 0.19039% |
| unclassified_Chloroflexi | 0.06870% | 0.09645% | 0.07104% | 0.15791% |
| *Dechloromonas* | 0.00536% | 0.00657% | 0.11943% | 0.15094% |
| *Bifidobacterium* | 0.06685% | 0.11957% | 0.06984% | 0.13434% |
| *Plesiomonas* | 0.00972% | 0.01002% | 0.11219% | 0.14413% |
| *Lactiplantibacillus* | 0.07389% | 0.06938% | 0.05804% | 0.06443% |
| *Malaciobacter* | 0.00436% | 0.01020% | 0.11246% | 0.24891% |
| unclassified_Intrasporangiaceae | 0.13237% | 0.08456% | 0.00965% | 0.00997% |
| *MND1* | 0.07104% | 0.13387% | 0.05791% | 0.14418% |
| unclassified_Xanthobacteraceae | 0.08076% | 0.14681% | 0.04986% | 0.08699% |
| *Jeotgalibacillus* | 0.02932% | 0.04234% | 0.08981% | 0.06765% |
| unclassified_Guggenheimella | 0.13706% | 0.17659% | 0.00094% | 0.00142% |
| Cylindrospermopsis_CRJ1 | 0.00034% | 0.00095% | 0.10442% | 0.18180% |
| *Proteocatella* | 0.08327% | 0.11997% | 0.03807% | 0.03961% |
| unclassified_Bacteroidia | 0.04675% | 0.13114% | 0.06581% | 0.10086% |
| *Planococcus* | 0.06535% | 0.05145% | 0.04745% | 0.06168% |
| C1_B045 | 0.05948% | 0.11075% | 0.05161% | 0.10058% |
| unclassified_Cryomorphaceae | 0.02413% | 0.04279% | 0.07748% | 0.12664% |
| Planktothrix_NIVA_CYA_15 | 0.00000% | 0.00000% | 0.09611% | 0.18745% |
| unclassified_Methylophilaceae | 0.00285% | 0.00647% | 0.09356% | 0.10582% |
| *Morganella* | 0.04876% | 0.04144% | 0.05670% | 0.07558% |
| unclassified_Vicinamibacterales | 0.05462% | 0.10005% | 0.05134% | 0.10852% |
| unclassified_Steroidobacteraceae | 0.06283% | 0.05475% | 0.04450% | 0.03858% |
| *Ligilactobacillus* | 0.06669% | 0.10059% | 0.04128% | 0.07061% |
| unclassified_Kineosporiaceae | 0.11645% | 0.16055% | 0.00054% | 0.00094% |
| *Photobacterium* | 0.01039% | 0.02008% | 0.08525% | 0.06593% |
| CL500_29_marine_group | 0.05512% | 0.03938% | 0.04825% | 0.02846% |
| unclassified_Mitochondria | 0.01324% | 0.01275% | 0.08109% | 0.07297% |
| unclassified_Blastocatellaceae | 0.05881% | 0.11019% | 0.04021% | 0.04097% |
| unclassified_A0839 | 0.00050% | 0.00069% | 0.08659% | 0.06678% |
| unclassified_Xanthomonadales | 0.00000% | 0.00000% | 0.08605% | 0.05592% |
| *Rothia* | 0.08160% | 0.07444% | 0.02037% | 0.02813% |
| uncultured_Chloroflexi_bacterium | 0.03602% | 0.06757% | 0.05616% | 0.13821% |
| unclassified_Rhodospirillales | 0.04859% | 0.03868% | 0.04343% | 0.04007% |
| unclassified_Microbacteriaceae | 0.06367% | 0.05421% | 0.03003% | 0.01887% |
| *Dysgonomonas* | 0.05982% | 0.04907% | 0.03311% | 0.04064% |
| *Phascolarctobacterium* | 0.03033% | 0.04972% | 0.05603% | 0.09133% |
| *Aerococcus* | 0.03552% | 0.06650% | 0.05147% | 0.05264% |
| uncultured_beta_proteobacterium | 0.00000% | 0.00000% | 0.07895% | 0.06732% |
| *Enhydrobacter* | 0.03318% | 0.02709% | 0.05228% | 0.07146% |
| unclassified_Flavobacteriales | 0.04976% | 0.06993% | 0.03780% | 0.04632% |
| *Gaiella* | 0.04457% | 0.07804% | 0.04035% | 0.09227% |
| *Ketobacter* | 0.04557% | 0.09801% | 0.03941% | 0.04188% |
| unclassified_Rhizobiales_Incertae_Sedis | 0.05714% | 0.05091% | 0.03003% | 0.03714% |
| *Lewinella* | 0.00000% | 0.00000% | 0.07506% | 0.07997% |
| *Brevibacterium* | 0.05211% | 0.07704% | 0.03257% | 0.03448% |
| *Haliscomenobacter* | 0.00017% | 0.00047% | 0.07211% | 0.06808% |
| *Microbacterium* | 0.04792% | 0.03563% | 0.03391% | 0.03774% |
| *Faecalibaculum* | 0.05378% | 0.08408% | 0.02815% | 0.03783% |
| SH3_11 | 0.00000% | 0.00000% | 0.07118% | 0.11818% |
| *Peredibacter* | 0.00905% | 0.00819% | 0.06354% | 0.06892% |
| *Candidatus* Chloroploca | 0.00017% | 0.00047% | 0.06970% | 0.07130% |
| *Micrococcus* | 0.05982% | 0.07426% | 0.02185% | 0.01444% |
| unclassified_Microscillaceae | 0.03552% | 0.07122% | 0.03968% | 0.08721% |
| *Halomonas* | 0.01474% | 0.02107% | 0.05388% | 0.04893% |
| unclassified_Micavibrionales | 0.08109% | 0.13342% | 0.00000% | 0.00000% |
| *Brachybacterium* | 0.04892% | 0.05782% | 0.02520% | 0.02315% |
| Burkholderia_Caballeronia_Paraburkholderia | 0.05445% | 0.06646% | 0.02011% | 0.01978% |
| *Dietzia* | 0.03133% | 0.06049% | 0.03847% | 0.08774% |
| *Lactobacillus* | 0.04239% | 0.07604% | 0.02895% | 0.03418% |
| unclassified_Balneolaceae | 0.00000% | 0.00000% | 0.06273% | 0.09838% |
| *Roseomonas* | 0.01056% | 0.01152% | 0.05335% | 0.05315% |
| *Ellin6067* | 0.02044% | 0.05307% | 0.04531% | 0.08583% |
| *Lacihabitans* | 0.07339% | 0.06912% | 0.00121% | 0.00293% |
| uncultured_Chlorobi_bacterium | 0.00000% | 0.00000% | 0.05965% | 0.16146% |
| *Aquaspirillum* | 0.00067% | 0.00190% | 0.05844% | 0.08076% |
| unclassified_Candidatus_Magasanikbacteria | 0.00000% | 0.00000% | 0.05817% | 0.17464% |
| *Massilia* | 0.01776% | 0.02232% | 0.04316% | 0.04308% |
| *Lutibacter* | 0.03183% | 0.06300% | 0.03150% | 0.07728% |
| unclassified_Acidimicrobiia | 0.03787% | 0.07339% | 0.02641% | 0.06715% |
| unclassified_Moraxellaceae | 0.00000% | 0.00000% | 0.05616% | 0.09309% |
| *Cereibacter* | 0.06836% | 0.07648% | 0.00134% | 0.00190% |
| *Sulfurovum* | 0.03267% | 0.06299% | 0.02815% | 0.04600% |
| *Terrimicrobium* | 0.00117% | 0.00332% | 0.05321% | 0.07767% |
| *Oceanobacter* | 0.00034% | 0.00062% | 0.05375% | 0.04335% |
| *Dongia* | 0.03569% | 0.06520% | 0.02507% | 0.05417% |
| *Serratia* | 0.02647% | 0.01882% | 0.03230% | 0.02406% |
| *Empedobacter* | 0.00034% | 0.00095% | 0.05295% | 0.05132% |
| unclassified_Pseudomonadales | 0.03401% | 0.06452% | 0.02560% | 0.06242% |
| *Kocuria* | 0.05094% | 0.03834% | 0.01153% | 0.01778% |
| *Solirubrobacter* | 0.01810% | 0.03426% | 0.03659% | 0.10559% |
| Lachnospiraceae_NK4A136_group | 0.01726% | 0.02588% | 0.03700% | 0.03481% |
| unclassified_Rokubacteriales | 0.02379% | 0.04522% | 0.03177% | 0.08974% |
| Nodularia_PCC_9350 | 0.00000% | 0.00000% | 0.05053% | 0.08368% |
| unclassified_Hyphomonadaceae | 0.01860% | 0.04457% | 0.03525% | 0.09131% |
| [Agitococcus]_lubricus_group | 0.00017% | 0.00047% | 0.05000% | 0.06323% |
| *Parasutterella* | 0.01860% | 0.03879% | 0.03525% | 0.06572% |
| *Robiginitalea* | 0.01625% | 0.02270% | 0.03659% | 0.05030% |
| Defluviitaleaceae_UCG_011 | 0.00620% | 0.01239% | 0.04437% | 0.11201% |
| Sva0996_marine_group | 0.06015% | 0.08332% | 0.00107% | 0.00176% |
| unclassified_MWH_UniP1_aquatic_group | 0.00034% | 0.00062% | 0.04879% | 0.04212% |
| unclassified_IMCC26256 | 0.02547% | 0.06573% | 0.02788% | 0.05596% |
| Anabaena_XPORK15F | 0.00000% | 0.00000% | 0.04758% | 0.10225% |
| *Legionella* | 0.01307% | 0.02109% | 0.03713% | 0.04812% |
| *Chelativorans* | 0.01106% | 0.01981% | 0.03740% | 0.09265% |
| *Alloprevotella* | 0.03116% | 0.05933% | 0.02131% | 0.04557% |
| *Lentimicrobium* | 0.00335% | 0.00895% | 0.04343% | 0.07649% |
| *Gemella* | 0.01910% | 0.03178% | 0.03070% | 0.05807% |
| *Veillonella* | 0.03167% | 0.04428% | 0.02064% | 0.02302% |
| *Devosia* | 0.02999% | 0.05035% | 0.02198% | 0.04709% |
| *Ornithinimicrobium* | 0.04122% | 0.02577% | 0.01139% | 0.01136% |
| unclassified_Xanthomonadaceae | 0.01357% | 0.03012% | 0.03338% | 0.03150% |
| unclassified_Ruminococcaceae | 0.04273% | 0.07929% | 0.01005% | 0.01947% |
| *Streptomyces* | 0.00637% | 0.01240% | 0.03860% | 0.08548% |
| *Microvirga* | 0.01726% | 0.02617% | 0.02976% | 0.06033% |
| *Gemmobacter* | 0.00804% | 0.01023% | 0.03700% | 0.04431% |
| *Rubrobacter* | 0.03250% | 0.06061% | 0.01716% | 0.03050% |
| *Trichococcus* | 0.02463% | 0.05371% | 0.02319% | 0.03032% |
| unclassified_Burkholderiales | 0.01391% | 0.03201% | 0.03137% | 0.03326% |
| *Azospira* | 0.00067% | 0.00101% | 0.04155% | 0.03922% |
| *Pseudoxanthomonas* | 0.00402% | 0.00672% | 0.03793% | 0.04195% |
| uncultured_prokaryote | 0.01826% | 0.03628% | 0.02641% | 0.02844% |
| EUB33_2 | 0.00000% | 0.00000% | 0.04048% | 0.07960% |
| hgcI_clade | 0.00620% | 0.00915% | 0.03512% | 0.03307% |
| Methylobacterium_Methylorubrum | 0.00972% | 0.01638% | 0.03230% | 0.04472% |
| unclassified_Cyanobacteriales | 0.00084% | 0.00237% | 0.03914% | 0.06082% |
| *Blautia* | 0.03200% | 0.06053% | 0.01394% | 0.03486% |
| unclassified_Desulfobulbaceae | 0.01810% | 0.04131% | 0.02507% | 0.06457% |
| *Cephaloticoccus* | 0.00017% | 0.00047% | 0.03927% | 0.05771% |
| *Ideonella* | 0.00117% | 0.00282% | 0.03820% | 0.04360% |
| uncultured_gamma_proteobacterium | 0.01776% | 0.02491% | 0.02453% | 0.03740% |
| *Hyphomonas* | 0.01826% | 0.03843% | 0.02373% | 0.02058% |
| *Rubellimicrobium* | 0.02798% | 0.01858% | 0.01582% | 0.01650% |
| *Falsirhodobacter* | 0.03167% | 0.03383% | 0.01287% | 0.01069% |
| *Arenimonas* | 0.01927% | 0.02141% | 0.02265% | 0.02808% |
| unclassified_TRA3_20 | 0.01022% | 0.01821% | 0.02976% | 0.07198% |
| *Sediminibacterium* | 0.00017% | 0.00047% | 0.03767% | 0.06764% |
| BD1_7_clade | 0.00034% | 0.00095% | 0.03753% | 0.03725% |
| unclassified_Anaerolineaceae | 0.01324% | 0.01726% | 0.02654% | 0.05917% |
| *Steroidobacter* | 0.01977% | 0.03697% | 0.02091% | 0.03943% |
| unclassified_Lactobacillales | 0.02614% | 0.02499% | 0.01582% | 0.02045% |
| *Pelomonas* | 0.03133% | 0.01433% | 0.01166% | 0.01232% |
| *Iamia* | 0.01826% | 0.01679% | 0.02185% | 0.04215% |
| Prevotella_7 | 0.02631% | 0.04498% | 0.01541% | 0.03538% |
| *Silvanigrella* | 0.00034% | 0.00095% | 0.03619% | 0.05858% |
| uncultured_proteobacterium | 0.01994% | 0.03615% | 0.02024% | 0.03799% |
| unclassified_Subgroup_7 | 0.02027% | 0.04604% | 0.01984% | 0.03442% |
| uncultured_Solirubrobacter_sp. | 0.02027% | 0.03982% | 0.01984% | 0.03055% |
| *Acetoanaerobium* | 0.03150% | 0.03541% | 0.01019% | 0.02013% |
| *Yersinia* | 0.02346% | 0.01676% | 0.01622% | 0.02714% |
| *Ramlibacter* | 0.02932% | 0.06005% | 0.01139% | 0.01350% |
| *Micromonospora* | 0.01676% | 0.02235% | 0.02118% | 0.02810% |
| unclassified_NS11_12_marine_group | 0.00000% | 0.00000% | 0.03458% | 0.07190% |
| *Terrimonas* | 0.00620% | 0.01239% | 0.02909% | 0.04259% |
| *Paludibacter* | 0.00067% | 0.00143% | 0.03338% | 0.04541% |
| *Thiobacillus* | 0.01139% | 0.01877% | 0.02480% | 0.04796% |
| *Carboxylicivirga* | 0.00067% | 0.00190% | 0.03324% | 0.03944% |
| unclassified_Salsuginimonas_clara | 0.00235% | 0.00611% | 0.03177% | 0.03895% |
| uncultured_Candidatus_Rokubactera_bacterium | 0.03301% | 0.06286% | 0.00643% | 0.01362% |
| unclassified_Microtrichales | 0.04088% | 0.07465% | 0.00000% | 0.00000% |
| *Ahniella* | 0.00352% | 0.00995% | 0.02989% | 0.04707% |
| *Subdoligranulum* | 0.01927% | 0.04147% | 0.01729% | 0.03846% |
| unclassified_Ilumatobacteraceae | 0.02044% | 0.03299% | 0.01622% | 0.03034% |
| unclassified_Lachnospiraceae | 0.02044% | 0.03434% | 0.01595% | 0.02909% |
| JGI_0001001_H03 | 0.02178% | 0.04639% | 0.01394% | 0.04223% |
| Microcystis_PCC_7914 | 0.01139% | 0.01818% | 0.02225% | 0.04545% |
| *Edaphobaculum* | 0.00369% | 0.00838% | 0.02828% | 0.08152% |
| unclassified_Chitinophagaceae | 0.03820% | 0.07020% | 0.00067% | 0.00114% |
| *Edaphobaculum* | 0.00314% | 0.00438% | 0.02864% | 0.07918% |
| *Serinicoccus* | 0.02681% | 0.03742% | 0.00965% | 0.01325% |
| unclassified_Microtrichales | 0.00000% | 0.00000% | 0.03108% | 0.06351% |
| uncultured_Acidobacteria_bacterium | 0.01324% | 0.02427% | 0.02037% | 0.05493% |
| unclassified_P13_46 | 0.00474% | 0.00888% | 0.02684% | 0.05039% |
| uncultured_soil_bacterium | 0.00922% | 0.02293% | 0.02319% | 0.06868% |
| unclassified_P13_46 | 0.01776% | 0.04018% | 0.01635% | 0.03772% |
| *Pseudohongiella* | 0.00105% | 0.00077% | 0.02929% | 0.05367% |
| *Pectobacterium* | 0.02346% | 0.01205% | 0.01126% | 0.02141% |
| *Crenobacter* | 0.00000% | 0.00000% | 0.03003% | 0.03929% |
| unclassified_Peptostreptococcaceae | 0.03100% | 0.05710% | 0.00509% | 0.01055% |
| *Crenobacter* | 0.02995% | 0.04475% | 0.00585% | 0.01337% |
| *Neisseria* | 0.02932% | 0.05396% | 0.00630% | 0.01125% |
| *Pectobacterium* | 0.00506% | 0.00758% | 0.02553% | 0.01852% |
| *Bryobacter* | 0.01743% | 0.04929% | 0.01555% | 0.02457% |
| *Aquitalea* | 0.00687% | 0.01012% | 0.02386% | 0.03340% |
| *Lysinibacillus* | 0.01407% | 0.02614% | 0.01796% | 0.01924% |
| *Pseudohongiella* | 0.01575% | 0.03229% | 0.01649% | 0.04838% |
| *Romboutsia* | 0.01709% | 0.02377% | 0.01541% | 0.02627% |
| *Candidatus* Nitrotoga | 0.00000% | 0.00000% | 0.02895% | 0.09062% |
| *Mycobacterium* | 0.01273% | 0.01583% | 0.01836% | 0.03525% |
| *Proteiniclasticum* | 0.01123% | 0.03121% | 0.01957% | 0.02230% |
| Erysipelotrichaceae_UCG_003 | 0.01659% | 0.03431% | 0.01515% | 0.04603% |
| unclassified_Desulfovibrionaceae | 0.00737% | 0.01033% | 0.02225% | 0.04421% |
| [Eubacterium]_eligens_group | 0.01273% | 0.03034% | 0.01743% | 0.05463% |
| *Facklamia* | 0.03385% | 0.03831% | 0.00027% | 0.00057% |
| *Dubosiella* | 0.00804% | 0.01720% | 0.02064% | 0.02845% |
| *Lachnospira* | 0.00838% | 0.02316% | 0.02024% | 0.05504% |
| unclassified_Bacilli | 0.00000% | 0.00000% | 0.02694% | 0.04348% |
| [Ruminococcus]_gnavus_group | 0.02161% | 0.04173% | 0.00938% | 0.01217% |
| unclassified_Myxococcota | 0.00989% | 0.02070% | 0.01877% | 0.04282% |
| [Eubacterium]_eligens_group | 0.00000% | 0.00000% | 0.02621% | 0.05673% |
| *Facklamia* | 0.00037% | 0.00081% | 0.02546% | 0.03423% |
| *Pedomicrobium* | 0.01776% | 0.02993% | 0.01139% | 0.02734% |
| uncultured_delta_proteobacterium | 0.00017% | 0.00047% | 0.02507% | 0.02996% |
| Christensenellaceae_R_7_group | 0.02564% | 0.06368% | 0.00456% | 0.01041% |
| *Haematococcus_lacustris* | 0.02302% | 0.03438% | 0.00640% | 0.02022% |
| unclassified_Prevotellaceae | 0.01441% | 0.03382% | 0.01327% | 0.04012% |
| unclassified_Prevotellaceae | 0.00043% | 0.00122% | 0.02419% | 0.04718% |
| *Colidextribacter* | 0.01960% | 0.03509% | 0.00885% | 0.01850% |
| *Aeromicrobium* | 0.01793% | 0.03692% | 0.01019% | 0.02989% |
| *Methylocystis* | 0.01324% | 0.02413% | 0.01367% | 0.01587% |
| *Mitsuaria* | 0.00535% | 0.00798% | 0.01988% | 0.02760% |
| *Acidibacter* | 0.01491% | 0.03395% | 0.01220% | 0.02135% |
| *Mitsuaria* | 0.01491% | 0.02245% | 0.01193% | 0.02133% |
| unclassified_Nocardioidaceae | 0.02178% | 0.01835% | 0.00643% | 0.00811% |
| Haematococcus_lacustris | 0.00000% | 0.00000% | 0.02373% | 0.03239% |
| unclassified_Paracaedibacteraceae | 0.01754% | 0.02183% | 0.00963% | 0.02989% |
| *Agathobacter* | 0.01190% | 0.02622% | 0.01394% | 0.03724% |
| uncultured_delta_proteobacterium | 0.02000% | 0.02414% | 0.00739% | 0.02309% |
| *Cutibacterium* | 0.01910% | 0.01303% | 0.00791% | 0.01147% |
| unclassified_B1_7BS | 0.01340% | 0.02952% | 0.01247% | 0.03314% |
| unclassified_Desulfocapsaceae | 0.01106% | 0.01517% | 0.01429% | 0.02501% |
| unclassified_Desulfocapsaceae | 0.00268% | 0.00491% | 0.02091% | 0.02647% |
| *Sneathiella* | 0.01087% | 0.02013% | 0.01430% | 0.03631% |
| unclassified_Paracaedibacteraceae | 0.00017% | 0.00047% | 0.02279% | 0.03128% |
| *Sneathiella* | 0.00402% | 0.00668% | 0.01970% | 0.03807% |
| *Bdellovibrio* | 0.00369% | 0.00794% | 0.01930% | 0.01532% |
| *Bauldia* | 0.00519% | 0.01221% | 0.01810% | 0.05581% |
| *Haoranjiania* | 0.01329% | 0.01839% | 0.01157% | 0.01741% |
| *Rhizorhapis* | 0.01525% | 0.01777% | 0.00992% | 0.01941% |
| *Cavicella* | 0.02721% | 0.03068% | 0.00033% | 0.00105% |
| *Haoranjiania* | 0.00754% | 0.01385% | 0.01595% | 0.02004% |
| *Fusicatenibacter* | 0.01257% | 0.02424% | 0.01180% | 0.02970% |
| *Mariniradius* | 0.02487% | 0.03711% | 0.00191% | 0.00604% |
| *Klebsiella* | 0.01785% | 0.01132% | 0.00747% | 0.00756% |
| *Woeseia* | 0.01391% | 0.02804% | 0.01059% | 0.02371% |
| *Mariniradius* | 0.00000% | 0.00000% | 0.02171% | 0.03371% |
| unclassified_Saccharimonadaceae | 0.02597% | 0.05142% | 0.00094% | 0.00179% |
| unclassified_Saccharimonadaceae | 0.00063% | 0.00120% | 0.02113% | 0.04874% |
| unclassified_GWA2_38_13b | 0.00000% | 0.00000% | 0.02158% | 0.02278% |
| *Klebsiella* | 0.00603% | 0.00447% | 0.01676% | 0.01145% |
| *Blastococcus* | 0.01743% | 0.03348% | 0.00751% | 0.01866% |
| *Cavicella* | 0.00000% | 0.00000% | 0.02145% | 0.02710% |
| *Woeseia* | 0.00389% | 0.00893% | 0.01794% | 0.03072% |
| unclassified_SJA_15 | 0.00460% | 0.00707% | 0.01699% | 0.02892% |
| unclassified_GWA2_38_13b | 0.02477% | 0.02294% | 0.00083% | 0.00263% |
| unclassified_Hyphomicrobiaceae | 0.00030% | 0.00061% | 0.02030% | 0.04301% |
| *Ileibacterium* | 0.01441% | 0.02729% | 0.00898% | 0.02305% |
| *Desulfopila* | 0.00268% | 0.00758% | 0.01836% | 0.01953% |
| *Marinomonas* | 0.00879% | 0.01083% | 0.01345% | 0.04197% |
| unclassified_SJA_15 | 0.01474% | 0.02934% | 0.00844% | 0.01594% |
| *Reyranella* | 0.00117% | 0.00231% | 0.01930% | 0.03497% |
| *Desulfopila* | 0.01793% | 0.01918% | 0.00588% | 0.01285% |
| uncultured_Bacteroidetes_Chlorobi_group_bacterium | 0.00469% | 0.00664% | 0.01635% | 0.03406% |
| unclassified_Candidatus_Nomurabacteria_bacterium_RIFCSPLOWO2_01_FULL_36_10b | 0.00658% | 0.01457% | 0.01459% | 0.03804% |
| *Chthonobacter* | 0.01139% | 0.02337% | 0.01072% | 0.02302% |
| *Roseicyclus* | 0.01642% | 0.02106% | 0.00670% | 0.00829% |
| *Marinomonas* | 0.00017% | 0.00047% | 0.01957% | 0.04020% |
| *Flavitalea* | 0.00771% | 0.01630% | 0.01354% | 0.03703% |
| uncultured_Bacteroidales_bacterium | 0.00972% | 0.01822% | 0.01180% | 0.02111% |
| *Chthonobacter* | 0.00369% | 0.00424% | 0.01659% | 0.03032% |
| Subgroup_23 | 0.01195% | 0.03347% | 0.00989% | 0.01685% |
| *Magnetospirillum* | 0.00000% | 0.00000% | 0.01944% | 0.03752% |
| *Methyloceanibacter* | 0.00938% | 0.01828% | 0.01193% | 0.02798% |
| *Methyloceanibacter* | 0.00359% | 0.00960% | 0.01651% | 0.02962% |
| *Roseicyclus* | 0.00635% | 0.00893% | 0.01424% | 0.01861% |
| *Truepera* | 0.02362% | 0.03591% | 0.00040% | 0.00065% |
| *Novispirillum* | 0.00000% | 0.00000% | 0.01930% | 0.02621% |
| Subgroup_23 | 0.01156% | 0.01940% | 0.01005% | 0.02818% |
| unclassified_Hyphomicrobiaceae | 0.01290% | 0.03595% | 0.00898% | 0.02793% |
| unclassified_Candidatus_Nomurabacteria_bacterium_RIFCSPLOWO2_01_FULL_36_10b | 0.00000% | 0.00000% | 0.01930% | 0.03578% |
| *Ileibacterium* | 0.00183% | 0.00453% | 0.01778% | 0.02922% |
| unclassified_Gammaproteobacteria | 0.02138% | 0.05037% | 0.00212% | 0.00518% |
| unclassified_Gammaproteobacteria | 0.00067% | 0.00124% | 0.01863% | 0.04445% |
| *Magnetospirillum* | 0.02083% | 0.04377% | 0.00241% | 0.00762% |
| unclassified_Methyloligellaceae | 0.00720% | 0.01984% | 0.01327% | 0.03613% |
| *Nitrospira* | 0.01508% | 0.02865% | 0.00684% | 0.01938% |
| *Novispirillum* | 0.02362% | 0.02735% | 0.00000% | 0.00000% |
| *Acanthopleuribacter* | 0.02296% | 0.06494% | 0.00020% | 0.00065% |
| Lachnospiraceae_UCG_004 | 0.00936% | 0.02617% | 0.01105% | 0.02109% |
| uncultured_Bacteroidetes_Chlorobi_group_bacterium | 0.01895% | 0.03341% | 0.00334% | 0.00546% |
| *Rhodococcus* | 0.01877% | 0.04626% | 0.00349% | 0.00569% |
| *Ignavibacterium* | 0.01227% | 0.01535% | 0.00861% | 0.02646% |
| *Ignavibacterium* | 0.00034% | 0.00095% | 0.01810% | 0.02647% |
| *Aquimonas* | 0.00017% | 0.00047% | 0.01823% | 0.01693% |
| *Halioglobus* | 0.00601% | 0.01508% | 0.01351% | 0.02530% |
| unclassified_Geminicoccaceae | 0.01676% | 0.03529% | 0.00469% | 0.00946% |
| Candidatus_Omnitrophus | 0.01488% | 0.01819% | 0.00618% | 0.01885% |
| *Paraprevotella* | 0.00000% | 0.00000% | 0.01799% | 0.04082% |
| Lachnospiraceae_UCG_004 | 0.00653% | 0.01505% | 0.01273% | 0.02662% |
| *Acanthopleuribacter* | 0.00017% | 0.00047% | 0.01783% | 0.05638% |
| *Terasakiella* | 0.00000% | 0.00000% | 0.01783% | 0.04894% |
| *Lachnoclostridium* | 0.01257% | 0.02405% | 0.00777% | 0.02276% |
| [Desulfobacterium]_catecholicum_group | 0.00000% | 0.00000% | 0.01783% | 0.02311% |
| *Rhodoplanes* | 0.01676% | 0.03307% | 0.00442% | 0.00975% |
| *Candidatus* Omnitrophus | 0.00034% | 0.00095% | 0.01743% | 0.02075% |
| *Paraprevotella* | 0.00687% | 0.01943% | 0.01220% | 0.03857% |
| [Desulfobacterium]_catecholicum_group | 0.01411% | 0.01861% | 0.00631% | 0.01996% |
| *Terasakiella* | 0.00297% | 0.00364% | 0.01520% | 0.04806% |
| uncultured_Actinomycetales_bacterium | 0.01910% | 0.03241% | 0.00228% | 0.00390% |
| *Rhodococcus* | 0.00372% | 0.00575% | 0.01454% | 0.03974% |
| *Flavonifractor* | 0.01709% | 0.03434% | 0.00375% | 0.01187% |
| uncultured_actinobacterium | 0.00117% | 0.00332% | 0.01649% | 0.03446% |
| *Halioglobus* | 0.01005% | 0.02225% | 0.00938% | 0.01919% |
| unclassified_0319_6G20 | 0.00000% | 0.00000% | 0.01689% | 0.03452% |
| unclassified_Parcubacteria_group_bacterium_GW2011_GWD2_42_14 | 0.00000% | 0.00000% | 0.01676% | 0.04305% |
| *Hungatella* | 0.00507% | 0.01397% | 0.01265% | 0.01846% |
| *Anaerostipes* | 0.00335% | 0.00948% | 0.01394% | 0.03011% |
| unclassified_Actinobacteriota | 0.01709% | 0.03372% | 0.00281% | 0.00594% |
| *Ruminococcus* | 0.01022% | 0.02732% | 0.00818% | 0.01738% |
| *Roseimarinus* | 0.00017% | 0.00047% | 0.01622% | 0.01439% |
| *Roseimarinus* | 0.01699% | 0.01540% | 0.00275% | 0.00809% |
| unclassified_0319_6G20 | 0.00670% | 0.01274% | 0.01096% | 0.03467% |
| *Altererythrobacter* | 0.01558% | 0.04193% | 0.00375% | 0.00809% |
| unclassified_Anaerolineae | 0.01307% | 0.02471% | 0.00563% | 0.01345% |
| unclassified_Parcubacteria_group_bacterium_GW2011_GWD2_42_14 | 0.00382% | 0.00953% | 0.01296% | 0.04097% |
| *Desulfomicrobium* | 0.01056% | 0.02932% | 0.00724% | 0.01614% |
| *Hungatella* | 0.00888% | 0.01404% | 0.00858% | 0.01796% |
| *Spiroplasma* | 0.00000% | 0.00000% | 0.01568% | 0.04772% |
| *Ruminococcus* | 0.00996% | 0.01888% | 0.00760% | 0.02250% |
| *Spiroplasma* | 0.01923% | 0.05262% | 0.00013% | 0.00042% |
| unclassified_Rhodanobacteraceae | 0.00905% | 0.01677% | 0.00818% | 0.01409% |
| *Lechevalieria* | 0.00687% | 0.01786% | 0.00978% | 0.02076% |
| *Lechevalieria* | 0.00374% | 0.00692% | 0.01194% | 0.02477% |
| *Collinsella* | 0.00318% | 0.00900% | 0.01180% | 0.02302% |
| [Anaerorhabdus]_furcosa_group | 0.00235% | 0.00558% | 0.01247% | 0.01782% |
| *Silanimonas* | 0.00000% | 0.00000% | 0.01394% | 0.01655% |
| unclassified_Oxalobacteraceae | 0.01139% | 0.01883% | 0.00442% | 0.00701% |
| *Propionigenium* | 0.00000% | 0.00000% | 0.01354% | 0.04281% |
| *Kribbella* | 0.00010% | 0.00030% | 0.01325% | 0.02347% |
| Coriobacteriaceae_UCG_002 | 0.00535% | 0.01067% | 0.00904% | 0.01826% |
| unclassified_Sutterellaceae | 0.01156% | 0.01299% | 0.00402% | 0.00956% |
| Coriobacteriaceae_UCG_002 | 0.00419% | 0.00815% | 0.00978% | 0.01894% |
| *Haliangium* | 0.00570% | 0.01456% | 0.00844% | 0.02624% |
| unclassified_Rhizobiales | 0.00251% | 0.00606% | 0.01099% | 0.03476% |
| *Kribbella* | 0.01089% | 0.02237% | 0.00416% | 0.01268% |
| *Desulfovibrio* | 0.01139% | 0.02808% | 0.00362% | 0.01011% |
| Rikenellaceae_RC9_gut_group | 0.00704% | 0.01784% | 0.00697% | 0.01845% |
| unclassified_[Eubacterium]_coprostanoligenes_group | 0.00989% | 0.02689% | 0.00469% | 0.01437% |
| uncultured_Firmicutes_bacterium | 0.01005% | 0.01861% | 0.00442% | 0.00937% |
| unclassified_Pedosphaeraceae | 0.01357% | 0.02777% | 0.00161% | 0.00509% |
| unclassified_Micropepsaceae | 0.00318% | 0.00848% | 0.00978% | 0.03094% |
| unclassified_Azospirillales | 0.00620% | 0.01214% | 0.00697% | 0.02112% |
| *Limosilactobacillus* | 0.00268% | 0.00758% | 0.00965% | 0.02912% |
| P3OB_42 | 0.00469% | 0.00672% | 0.00777% | 0.01851% |
| *Xanthobacter* | 0.00549% | 0.01554% | 0.00693% | 0.02192% |
| *Saccharomonospora* | 0.00603% | 0.01501% | 0.00630% | 0.01728% |
| *Providencia* | 0.00620% | 0.00848% | 0.00603% | 0.00916% |
| *Leucobacter* | 0.01089% | 0.01167% | 0.00201% | 0.00348% |
| unclassified_Oscillospiraceae | 0.00586% | 0.01659% | 0.00590% | 0.01116% |
| *Xanthobacter* | 0.00821% | 0.02322% | 0.00389% | 0.01229% |
| *Roseburia* | 0.00419% | 0.00776% | 0.00657% | 0.01485% |
| *Phenylobacterium* | 0.00553% | 0.00896% | 0.00550% | 0.01090% |
| *Thermomonas* | 0.00117% | 0.00220% | 0.00858% | 0.01873% |
| *Actinotalea* | 0.00922% | 0.01200% | 0.00214% | 0.00633% |
| *Cellulosimicrobium* | 0.01005% | 0.01214% | 0.00121% | 0.00337% |
| *Bosea* | 0.00821% | 0.01497% | 0.00241% | 0.00327% |
| *Nordella* | 0.01089% | 0.02207% | 0.00027% | 0.00085% |
| *Caulobacter* | 0.00201% | 0.00336% | 0.00737% | 0.01198% |
| unclassified_Frankiales | 0.00955% | 0.02701% | 0.00121% | 0.00381% |
| *Butyricicoccus* | 0.00821% | 0.01521% | 0.00228% | 0.00632% |
| *Macellibacteroides* | 0.00000% | 0.00000% | 0.00831% | 0.01391% |
| *Enterorhabdus* | 0.00905% | 0.02559% | 0.00094% | 0.00297% |
| [Ruminococcus]_torques_group | 0.00385% | 0.01090% | 0.00509% | 0.01051% |
| unclassified_WX65 | 0.00989% | 0.01722% | 0.00027% | 0.00085% |
| *Pseudolabrys* | 0.00653% | 0.01223% | 0.00268% | 0.00802% |
| uncultured_Desulfovirga_sp. | 0.00369% | 0.00939% | 0.00469% | 0.01484% |
| unclassified_SBR1031 | 0.00720% | 0.01782% | 0.00188% | 0.00548% |
| *Thauera* | 0.00268% | 0.00758% | 0.00550% | 0.01338% |
| uncultured_Alphaproteobacteria_bacterium | 0.00922% | 0.02251% | 0.00000% | 0.00000% |
| unclassified_Acidobacteriaceae__Subgroup_1 | 0.00922% | 0.02606% | 0.00000% | 0.00000% |
| *Sphingoaurantiacus* | 0.00771% | 0.01443% | 0.00121% | 0.00256% |
| *Coprococcus* | 0.00838% | 0.02209% | 0.00027% | 0.00085% |
| *Dorea* | 0.00067% | 0.00190% | 0.00643% | 0.02035% |
| Clostridium_sensu_stricto_11 | 0.00134% | 0.00379% | 0.00576% | 0.01596% |
| unclassified_KF_JG30_B3 | 0.00251% | 0.00478% | 0.00456% | 0.01116% |
| *Akkermansia* | 0.00771% | 0.01630% | 0.00013% | 0.00042% |
| *Achromobacter* | 0.00687% | 0.00902% | 0.00067% | 0.00170% |
| *Anaerovorax* | 0.00486% | 0.00982% | 0.00228% | 0.00586% |
| *Georgenia* | 0.00737% | 0.01367% | 0.00013% | 0.00042% |
| *Proteus* | 0.00184% | 0.00328% | 0.00416% | 0.01000% |
| UCG_002 | 0.00570% | 0.01611% | 0.00094% | 0.00297% |
| unclassified_Caulobacteraceae | 0.00134% | 0.00379% | 0.00442% | 0.01399% |
| *Holdemanella* | 0.00503% | 0.01422% | 0.00134% | 0.00340% |
| *Nocardioides* | 0.00637% | 0.01181% | 0.00027% | 0.00085% |
| *Muribaculum* | 0.00620% | 0.01267% | 0.00013% | 0.00042% |
| uncultured_bacterium_gp6 | 0.00637% | 0.01311% | 0.00000% | 0.00000% |
| unclassified_Acidobacteriales | 0.00235% | 0.00663% | 0.00308% | 0.00975% |
| unclassified_OPB41 | 0.00000% | 0.00000% | 0.00496% | 0.01568% |
| unclassified_Subgroup_17 | 0.00335% | 0.00948% | 0.00228% | 0.00593% |
| Lachnospiraceae_UCG_006 | 0.00385% | 0.01090% | 0.00161% | 0.00345% |
| *Desulfobotulus* | 0.00000% | 0.00000% | 0.00469% | 0.01437% |
| *Opitutus* | 0.00101% | 0.00284% | 0.00375% | 0.01141% |
| uncultured_forest_soil_bacterium | 0.00553% | 0.01156% | 0.00000% | 0.00000% |
| Subgroup_10 | 0.00168% | 0.00474% | 0.00281% | 0.00800% |
| *Sporosarcina* | 0.00084% | 0.00100% | 0.00349% | 0.00713% |
| *Candidatus* Saccharimonas | 0.00117% | 0.00332% | 0.00295% | 0.00886% |
| *Dialister* | 0.00101% | 0.00284% | 0.00308% | 0.00792% |
| IMCC26207 | 0.00117% | 0.00332% | 0.00281% | 0.00798% |
| *Oscillibacter* | 0.00000% | 0.00000% | 0.00362% | 0.00866% |
| unclassified_Clostridia_UCG_014 | 0.00134% | 0.00287% | 0.00228% | 0.00593% |
| unclassified_Rikenellaceae | 0.00000% | 0.00000% | 0.00335% | 0.01060% |
| *Alcaligenes* | 0.00000% | 0.00000% | 0.00295% | 0.00394% |
| Prevotellaceae_UCG_001 | 0.00151% | 0.00427% | 0.00174% | 0.00551% |
| unclassified_Roseiflexaceae | 0.00000% | 0.00000% | 0.00281% | 0.00417% |
| *Candidatus* Udaeobacter | 0.00352% | 0.00652% | 0.00000% | 0.00000% |
| *Sphingopyxis* | 0.00000% | 0.00000% | 0.00268% | 0.00603% |
| *Acidipila Silvibacterium* | 0.00318% | 0.00900% | 0.00000% | 0.00000% |
| *Rhodanobacter* | 0.00117% | 0.00332% | 0.00147% | 0.00466% |
| *Luteimonas* | 0.00218% | 0.00417% | 0.00040% | 0.00090% |
| Prevotellaceae_Ga6A1_group | 0.00268% | 0.00758% | 0.00000% | 0.00000% |
| *Soehngenia* | 0.00251% | 0.00711% | 0.00000% | 0.00000% |
| *Megamonas* | 0.00235% | 0.00440% | 0.00000% | 0.00000% |
| *Polynucleobacter* | 0.00218% | 0.00564% | 0.00000% | 0.00000% |
| *Pediococcus* | 0.00218% | 0.00616% | 0.00000% | 0.00000% |
| GCA_900066575 | 0.00201% | 0.00569% | 0.00000% | 0.00000% |
| Incertae_Sedis | 0.00000% | 0.00000% | 0.00161% | 0.00509% |
| *Helicobacter* | 0.00151% | 0.00427% | 0.00040% | 0.00127% |
| *Pusillimonas* | 0.00117% | 0.00332% | 0.00054% | 0.00170% |
| Clostridium_sensu_stricto_1 | 0.00184% | 0.00521% | 0.00000% | 0.00000% |
| unclassified_SAR202_clade | 0.00151% | 0.00324% | 0.00000% | 0.00000% |
| *Monoglobus* | 0.00134% | 0.00379% | 0.00000% | 0.00000% |
| [Eubacterium]_xylanophilum_group | 0.00134% | 0.00379% | 0.00000% | 0.00000% |
| *Allobaculum* | 0.00000% | 0.00000% | 0.00067% | 0.00212% |
| TM7x | 0.00084% | 0.00237% | 0.00000% | 0.00000% |
| *Terrisporobacter* | 0.00084% | 0.00159% | 0.00000% | 0.00000% |
| unclassified_Alphaproteobacteria | 0.00000% | 0.00000% | 0.00067% | 0.00212% |
| *Alistipes* | 0.00050% | 0.00142% | 0.00013% | 0.00042% |
| *Candidatus* Solibacter | 0.00067% | 0.00190% | 0.00000% | 0.00000% |
| *Catenibacterium* | 0.00000% | 0.00000% | 0.00054% | 0.00170% |
| alphaI_cluster | 0.00000% | 0.00000% | 0.00040% | 0.00127% |
| unclassified_Christensenellaceae | 0.00000% | 0.00000% | 0.00013% | 0.00042% |

**Supplementary Table 2 The relative abundance of fungal taxa in the hemolymph of WMS and Healthy crabs at the genus level**

|  | Healthy_hemolymph | | WMS_hemolymph | |
| --- | --- | --- | --- | --- |
| Fungal Genera | mean | sd | mean | sd |
| *Vanrija* | 64.04292% | 5.86162% | 64.81966% | 8.92066% |
| *Aspergillus* | 6.17749% | 1.34348% | 4.46414% | 2.44753% |
| *Kurtzmaniella* | 1.74567% | 0.48809% | 3.90265% | 1.11146% |
| unclassified_Ascomycota | 1.94052% | 0.83604% | 2.99757% | 2.19618% |
| *Candida* | 1.85509% | 1.60303% | 2.23336% | 0.52744% |
| *Clavispora* | 1.41093% | 0.69866% | 1.74311% | 0.75256% |
| *Fusarium* | 1.75010% | 0.77932% | 1.62599% | 0.85611% |
| *Wickerhamomyces* | 1.47541% | 1.26832% | 1.60544% | 0.99258% |
| *Teunomyces* | 4.11522% | 1.09965% | 1.56114% | 0.93806% |
| unclassified_Basidiomycota | 0.88762% | 0.23232% | 1.37677% | 1.10722% |
| *Tausonia* | 1.02147% | 0.38565% | 1.09922% | 0.43140% |
| unclassified_Onygenales | 0.88506% | 0.35389% | 0.86278% | 0.32038% |
| unclassified_Leotiomycetes | 0.92882% | 0.26201% | 0.84223% | 0.34398% |
| *Geotrichum* | 0.84223% | 0.63127% | 0.84163% | 0.52100% |
| *Penicillium* | 0.52401% | 0.25195% | 0.79434% | 0.49980% |
| *Cladosporium* | 1.15975% | 0.84297% | 0.72670% | 0.44774% |
| *Fusicolla* | 0.39597% | 0.11442% | 0.56388% | 0.25561% |
| *Acaulium* | 0.57382% | 0.11832% | 0.45154% | 0.53081% |
| unclassified_Chaetomiaceae | 0.31822% | 0.25567% | 0.39128% | 0.26064% |
| *Oidiodendron* | 0.27725% | 0.24958% | 0.34539% | 0.22462% |
| *Pseudogymnoascus* | 0.61735% | 0.38845% | 0.34060% | 0.25028% |
| *Trichoderma* | 0.24373% | 0.14237% | 0.29551% | 0.22812% |
| *Gliomastix* | 0.26142% | 0.16110% | 0.29152% | 0.26864% |
| unclassified_Pleosporales | 0.26445% | 0.22642% | 0.27017% | 0.20839% |
| unclassified_Pseudeurotiaceae | 0.40063% | 0.30639% | 0.25780% | 0.14733% |
| *Unclassified* | 0.08730% | 0.12338% | 0.17718% | 0.34022% |
| *Acremonium* | 0.17948% | 0.19398% | 0.17279% | 0.12794% |
| *Exophiala* | 0.25071% | 0.19353% | 0.16422% | 0.08257% |
| *Phialemoniopsis* | 0.44556% | 0.45516% | 0.15324% | 0.08163% |
| *Psathyrella* | 0.00396% | 0.00206% | 0.15105% | 0.34936% |
| *Cercospora* | 0.00279% | 0.00234% | 0.15065% | 0.36137% |
| *Ochroconis* | 0.11220% | 0.09152% | 0.14366% | 0.07118% |
| *Alternaria* | 0.20136% | 0.12379% | 0.13927% | 0.11825% |
| *Talaromyces* | 0.00163% | 0.00206% | 0.13089% | 0.20969% |
| *Atractiella* | 0.19298% | 0.11026% | 0.12910% | 0.07635% |
| *Mucor* | 0.01257% | 0.02669% | 0.12092% | 0.21430% |
| *Pseudallescheria* | 0.01816% | 0.03367% | 0.11493% | 0.17073% |
| *Monocillium* | 0.08753% | 0.05928% | 0.11114% | 0.11765% |
| *Pleotrichocladium* | 0.13758% | 0.16477% | 0.11054% | 0.16947% |
| unclassified_Dothideomycetes | 0.00140% | 0.00153% | 0.09159% | 0.23800% |
| unclassified_Didymellaceae | 0.18134% | 0.20998% | 0.08460% | 0.06025% |
| *Aureobasidium* | 0.24792% | 0.21689% | 0.07283% | 0.07604% |
| unclassified_Cucurbitariaceae | 0.06960% | 0.16162% | 0.06724% | 0.07312% |
| unclassified_Eurotiomycetes | 0.03655% | 0.05558% | 0.06624% | 0.09344% |
| *Pyrenochaeta* | 0.05680% | 0.08870% | 0.06525% | 0.07584% |
| *Kalmusia* | 0.00023% | 0.00057% | 0.06525% | 0.17263% |
| *Zygorhizidium* | 0.00070% | 0.00077% | 0.06385% | 0.12445% |
| unclassified_Sordariomycetes | 0.00023% | 0.00057% | 0.06285% | 0.11817% |
| *Pichia* | 0.06378% | 0.09885% | 0.05786% | 0.09178% |
| *Saccharomyces* | 0.18902% | 0.19292% | 0.05747% | 0.06560% |
| *Starmerella* | 0.03306% | 0.07360% | 0.04769% | 0.12494% |
| *Yarrowia* | 0.02374% | 0.02226% | 0.04649% | 0.04805% |
| *Trichocladium* | 0.07822% | 0.11741% | 0.04589% | 0.04413% |
| *Apiotrichum* | 0.09544% | 0.19272% | 0.04509% | 0.05887% |
| *Inocybe* | 0.00931% | 0.01467% | 0.04270% | 0.06587% |
| *Cutaneotrichosporon* | 0.03469% | 0.07949% | 0.03871% | 0.06199% |
| *Neocucurbitaria* | 0.00070% | 0.00171% | 0.03811% | 0.06272% |
| *Knufia* | 0.01373% | 0.03161% | 0.03492% | 0.09177% |
| *Botryotrichum* | 0.06262% | 0.09773% | 0.03292% | 0.04273% |
| *Vishniacozyma* | 0.03189% | 0.07608% | 0.03252% | 0.08298% |
| *Saitozyma* | 0.11174% | 0.11925% | 0.03133% | 0.04869% |
| unclassified_Phaeosphaeriaceae | 0.01467% | 0.03323% | 0.03093% | 0.07937% |
| *Xenomyrothecium* | 0.00000% | 0.00000% | 0.02913% | 0.05030% |
| *Keithomyces* | 0.00047% | 0.00072% | 0.02734% | 0.07232% |
| *Gymnoascus* | 0.00000% | 0.00000% | 0.02674% | 0.06890% |
| *Stellatospora* | 0.12687% | 0.10559% | 0.02634% | 0.04101% |
| *Westerdykella* | 0.04935% | 0.07823% | 0.02594% | 0.06618% |
| unclassified_Agaricomycetes | 0.00023% | 0.00057% | 0.02494% | 0.06476% |
| *Wallemia* | 0.00116% | 0.00163% | 0.02474% | 0.05614% |
| *Trechispora* | 0.02910% | 0.04350% | 0.02434% | 0.03346% |
| *Emericellopsis* | 0.02840% | 0.06618% | 0.02414% | 0.04336% |
| *Teichospora* | 0.05028% | 0.11975% | 0.02394% | 0.06212% |
| *Eutypella* | 0.00023% | 0.00057% | 0.02315% | 0.06062% |
| *Phaeosphaeria* | 0.00000% | 0.00000% | 0.02155% | 0.05518% |
| *Ambrosiozyma* | 0.00093% | 0.00169% | 0.02135% | 0.05649% |
| *Chalara* | 0.00070% | 0.00117% | 0.02075% | 0.03662% |
| unclassified_Dipodascaceae | 0.14503% | 0.21925% | 0.02075% | 0.00870% |
| *Dactylonectria* | 0.04749% | 0.07514% | 0.02015% | 0.04907% |
| *Purpureocillium* | 0.20788% | 0.20510% | 0.01696% | 0.02546% |
| *Hortaea* | 0.03725% | 0.09055% | 0.01516% | 0.04012% |
| *Zygosaccharomyces* | 0.03189% | 0.04843% | 0.01337% | 0.03230% |
| *Hygrocybe* | 0.01164% | 0.02783% | 0.01337% | 0.03234% |
| *Scopulariopsis* | 0.01862% | 0.04562% | 0.01337% | 0.03414% |
| *Debaryomyces* | 0.20066% | 0.36556% | 0.01317% | 0.01307% |
| *Humicola* | 0.04772% | 0.07798% | 0.01058% | 0.02798% |
| *Amauroascus* | 0.02980% | 0.07027% | 0.01058% | 0.01776% |
| *Yunnania* | 0.01932% | 0.04733% | 0.00998% | 0.02578% |
| *Gibberella* | 0.11663% | 0.19995% | 0.00878% | 0.01899% |
| *Bipolaris* | 0.01746% | 0.04277% | 0.00738% | 0.01892% |
| *Dioszegia* | 0.06634% | 0.10086% | 0.00678% | 0.01263% |
| *Lecanicillium* | 0.02258% | 0.05463% | 0.00299% | 0.00677% |
| *Pseudocercospora* | 0.07170% | 0.12644% | 0.00259% | 0.00234% |
| *Corticium* | 0.06052% | 0.13801% | 0.00180% | 0.00155% |
| *Dendryphion* | 0.06565% | 0.16080% | 0.00160% | 0.00204% |
| *Hannaella* | 0.03189% | 0.07470% | 0.00160% | 0.00149% |
| unclassified_Aspergillaceae | 0.02188% | 0.05360% | 0.00120% | 0.00149% |
| *Strelitziana* | 0.08217% | 0.13916% | 0.00100% | 0.00106% |
| *Colletotrichum* | 0.03631% | 0.08691% | 0.00100% | 0.00209% |
| unclassified_Nectriaceae | 0.02863% | 0.06810% | 0.00080% | 0.00110% |
| *Bacilliformis* | 0.02677% | 0.06489% | 0.00080% | 0.00110% |
| *Leptosphaeria* | 0.03352% | 0.07938% | 0.00060% | 0.00075% |
| unclassified_Sordariaceae | 0.05168% | 0.12590% | 0.00040% | 0.00106% |
| *Monascus* | 0.02631% | 0.06443% | 0.00040% | 0.00106% |
| unclassified_Lentitheciaceae | 0.08054% | 0.19593% | 0.00020% | 0.00053% |
| unclassified_Cylindrosympodiaceae | 0.02747% | 0.06728% | 0.00020% | 0.00053% |
| *Tuber* | 0.02677% | 0.06353% | 0.00020% | 0.00053% |
| *Phlyctochytrium* | 0.02305% | 0.04420% | 0.00020% | 0.00053% |
| *Paraphaeosphaeria* | 0.03492% | 0.08485% | 0.00000% | 0.00000% |
| *Paraphoma* | 0.02910% | 0.06923% | 0.00000% | 0.00000% |
| *Preussia* | 0.02887% | 0.07002% | 0.00000% | 0.00000% |

**Supplementary Table 3** **Sequence, taxonomy, and other information of isolated bacterial species**

| No. | Genus | *species* | score | coverage | identity | accession |
| --- | --- | --- | --- | --- | --- | --- |
| SYSUB030 | *Chryseomicrobium* | *Chryseomicrobium imtechense* | 1531 | 97% | 98.73% | NR_117419.1 |
| SYSUB031 | *Exiguobacterium* | *Exiguobacterium acetylicum* | 1757 | 99% | 99.79% | KT986087.1 |
| SYSUB028 | *Priestia* | *Priestia aryabhattai* | 2207 | 100% | 100% | OP457078.1 |
| SYSUB035 | *Staphylococcus* | *Staphylococcus epidermidis* | 1916 | 99% | 99.07% | FJ613565.1 |
| SYSUB011 | *Acinetobacter* | *Acinetobacter johnsonii* | 1676 | 100% | 100% | MT226917.1 |
| SYSUB013 | *Aeromonas* | *Aeromonas bivalvium* | 1831 | 99% | 99.70% | KF500920.1 |
| SYSUB014 | *Aeromonas* | *Aeromonas hydrophila* | 1877 | 98% | 99.14% | MT279533.1 |
| SYSUB015 | *Aeromonas* | *Aeromonas media* | 1832 | 98% | 98.56% | KC210759.1 |
| SYSUB018 | *Comamonas* | *Comamonas koreensis* | 1805 | 100% | 100% | MN889377.1 |
| SYSUB020 | *Enterobacter* | *Enterobacter ludwigii* | 1530 | 100% | 100% | MN330013.1 |
| SYSUB023 | *Proteus* | *Proteus penneri* | 1845 | 100% | 100% | LN809884.1 |
| SYSUB025 | *Pseudomonas* | *Pseudomonas putida* | 1760 | 98% | 100% | MN826550.1 |
| SYSUB007 | *Shewanella* | *Shewanella algae* | 1783 | 97% | 98.52% | AB205579.1 |
| SYSUB001 | *Shewanella* | *Shewanella chilikensis* | 1965 | 97% | 97.82% | NR_117772.1 |
| SYSUB009 | *Shewanella* | *Shewanella hafniensis* | 1698 | 100% | 100% | NR_041296.1 |
| SYSUB002 | *Shewanella* | *Shewanella putrefaciens* | 1448 | 100% | 100% | KX692893.1 |
| SYSUB003 | *Shewanella* | *Shewanella xiamenensis* | 1690 | 98% | 98.64% | HQ418492.1 |
| SYSUB026 | *Stutzerimonas* | *Stutzerimonas stutzeri* | 2141 | 100% | 100% | AB109011.1 |
| SYSUB004 | *Vibrio* | *Vibrio alginolyticus* | 1531 | 100% | 100% | MN180830.1 |
| SYSUB010 | *Vibrio* | *Vibrio azureus* | 1845 | 100% | 100% | MW996727.1 |
| SYSUB005 | *Vibrio* | *Vibrio fluvialis* | 1629 | 100% | 100% | MK294276.1 |
| SYSUB022 | *Vibrio* | *Vibrio neocaledonicus* | 2591 | 100% | 100% | OL584447.1 |
| SYSUB006 | *Vibrio* | *Vibrio parahaemolyticus* | 1740 | 99% | 99.17% | GQ205448.1 |

**Supplementary Table 4** **Sequence, taxonomy, and other information of isolated fungal species**

| No. | Genus | species | score | coverage | identity | accession |
| --- | --- | --- | --- | --- | --- | --- |
| SYSUF001 | *Cladosporium* | *Cladosporium cycadicola* | 990 | 100% | 100% | NR_156279.1 |
| SYSUF002 | *Curvularia* | *Curvularia petersonii* | 861 | 100% | 100% | OM809822.1 |
| SYSUF007 | *Debaryomyces* | *Debaryomyces hansenii* | 1114 | 99% | 99.67% | OM959374.1 |
| SYSUF003 | *Hortaea* | *Hortaea werneckii* | 942 | 99% | 99.42% | JX141367.1 |
| SYSUF008 | *Lodderomyces* | *Lodderomyces elongisporus* | 955 | 98% | 99.81% | KP674957.1 |
| SYSUF010 | *Meyerozyma* | *Meyerozyma guilliermondii* | 1037 | 98% | 99.30% | ON242325.1 |
| SYSUF005 | *Penicillium* | *Penicillium goetzii* | 1062 | 100% | 100% | NR_111820.1 |
| SYSUF004 | *Penicillium* | *Penicillium rubens* | 983 | 100% | 100% | MN413181.1 |
| SYSUF012 | *Simplicillium* | *Simplicillium sympodiophorum* | 1048 | 97% | 99.14% | NR_111027.1 |
| SYSUF013 | *Trichoderma* | *Trichoderma effusum* | 907 | 100% | 100% | NR_111833.1 |
| SYSUF014 | *Trichoderma* | *Trichoderma gamsii* | 1033 | 100% | 100% | NR_131317.1 |
| SYSUF015 | *Trichoderma* | *Trichoderma saturnisporum* | 1147 | 100% | 100% | NR_103704.1 |
| SYSUF011 | *Wickerhamomyces* | *Wickerhamomyces anomalus* | 1044 | 100% | 100% | NR_111210.1 |
| SYSUF017 | *Macrocybe* | *Macrocybe gigantea* | 1182 | 100% | 100% | KF360838.1 |

**Supplementary Table 5** **Distribution of the isolated bacteria in the hemolymph and muscle tissues of each WMS and Healthy crab**

|  | WMS 1 | | WMS 2 | | WMS 3 | | WMS 4 | | WMS 5 | | WMS 6 | | WMS 7 | | WMS 8 | | WMS 9 | | WMS 10 | |
| --- | --- | --- | --- | --- | --- | --- | --- | --- | --- | --- | --- | --- | --- | --- | --- | --- | --- | --- | --- | --- |
|  | Hem. | M | Hem. | M | Hem. | M | Hem. | M | Hem. | M | Hem. | M | Hem. | M | Hem. | M | Hem. | M | Hem. | M |
| *Acinetobacter johnsonii* | √ |  | √ |  | √ |  |  |  | √ |  | √ |  | √ |  | √ |  | √ |  | √ |  |
| *Aeromonas bivalvium* | √ |  |  |  | √ |  | √ |  |  |  | √ |  |  |  |  |  |  |  | √ |  |
| *Aeromonas hydrophila* |  |  |  |  |  |  |  |  |  |  |  |  |  |  |  |  |  |  |  |  |
| *Aeromonas media* |  |  |  |  |  |  |  |  |  |  |  |  |  |  |  |  |  |  |  |  |
| *Priestia aryabhattai* |  | √ |  | √ |  | √ |  |  |  |  |  |  |  | √ |  |  |  |  |  |  |
| *Chryseomicrobium imtechense* | √ |  | √ |  | √ |  |  |  | √ |  | √ |  | √ |  | √ |  | √ |  | √ |  |
| *Comamonas koreensis* | √ |  | √ |  |  |  | √ |  | √ |  | √ |  | √ |  |  |  |  |  |  |  |
| *Enterobacter ludwigii* |  |  |  |  |  |  |  |  |  |  |  |  |  |  |  |  |  |  |  |  |
| *Exiguobacterium acetylicum* |  |  |  |  |  |  |  |  |  |  |  |  |  |  |  |  |  |  |  |  |
| *Proteus penneri* | √ |  | √ |  |  |  | √ |  | √ |  | √ |  | √ |  |  |  |  |  |  |  |
| *Pseudomonas putida* |  |  |  |  |  |  |  |  |  |  |  |  |  |  |  |  |  |  |  |  |
| *Stutzerimonas stutzeri* | √ | √ | √ | √ |  | √ | √ |  | √ |  | √ |  | √ | √ |  |  |  | √ |  |  |
| *Shewanella algae* | √ |  | √ |  | √ |  | √ |  | √ |  | √ |  | √ |  | √ |  | √ |  | √ |  |
| *Shewanella chilikensis* | √ | √ | √ | √ | √ | √ |  | √ | √ | √ | √ | √ | √ |  | √ | √ | √ | √ | √ | √ |
| *Shewanella hafniensis* |  |  |  |  | √ |  | √ |  |  |  | √ |  |  |  |  |  |  |  |  |  |
| *Shewanella putrefaciens* | √ | √ |  | √ | √ | √ | √ | √ | √ | √ | √ | √ | √ |  |  | √ |  | √ | √ | √ |
| *Shewanella xiamenensis* | √ | √ | √ | √ | √ | √ |  | √ | √ | √ | √ | √ | √ | √ | √ | √ | √ | √ | √ | √ |
| *Staphylococcus epidermidis* |  | √ |  | √ |  | √ |  | √ |  |  |  |  |  | √ |  |  |  | √ |  | √ |
| *Vibrio alginolyticus* | √ | √ | √ | √ | √ | √ |  | √ | √ | √ | √ | √ | √ | √ | √ | √ | √ | √ | √ | √ |
| *Vibrio azureus* | √ |  |  |  | √ |  | √ |  |  |  | √ |  | √ |  | √ |  |  |  | √ |  |
| *Vibrio fluvialis* | √ | √ | √ | √ |  | √ | √ | √ | √ | √ | √ | √ | √ | √ |  | √ |  | √ | √ | √ |
| *Vibrio neocaledonicus* |  |  |  |  |  |  |  |  |  |  |  |  |  |  |  |  |  |  |  |  |
| *Vibrio parahaemolyticus* | √ | √ |  | √ | √ | √ |  | √ |  | √ | √ |  | √ | √ | √ | √ | √ | √ | √ | √ |
|  | Healthy 1 | | Healthy 2 | | Healthy 3 | | Healthy 4 | | Healthy 5 | | Healthy 6 | | Healthy 7 | | Healthy 8 | | Healthy 9 | | Healthy 10 | |
|  | Hem. | M | Hem. | M | Hem. | M | Hem. | M | Hem. | M | Hem. | M | Hem. | M | Hem. | M | Hem. | M | Hem. | M |
| *Acinetobacter johnsonii* |  |  |  |  | √ |  |  |  |  |  |  |  |  |  |  |  |  |  |  |  |
| *Aeromonas bivalvium* |  |  |  |  |  |  |  |  | √ |  |  |  |  |  |  |  |  |  |  |  |
| *Aeromonas hydrophila* |  |  |  |  |  |  |  |  |  | √ |  | √ |  |  |  |  | √ | √ |  |  |
| *Aeromonas media* |  |  |  |  |  |  |  |  |  |  |  |  |  |  |  | √ |  |  | √ | √ |
| *Priestia aryabhattai* |  |  |  |  |  |  |  |  |  |  |  |  |  |  |  |  |  |  |  |  |
| *Chryseomicrobium imtechense* |  |  |  |  |  |  |  |  |  |  |  |  |  |  |  |  |  |  |  |  |
| *Comamonas koreensis* |  |  |  |  |  |  |  |  |  |  |  |  |  |  |  |  |  |  |  |  |
| *Enterobacter ludwigii* |  |  | √ |  |  |  |  |  |  |  |  |  |  |  |  |  |  |  | √ |  |
| *Exiguobacterium acetylicum* | √ |  |  |  |  |  |  |  | √ |  |  |  |  |  |  |  |  |  |  |  |
| *Proteus penneri* |  |  |  |  |  |  |  |  |  |  |  |  |  |  |  |  |  |  |  |  |
| *Pseudomonas putida* |  |  | √ |  |  |  | √ |  |  |  | √ |  |  |  |  |  | √ |  |  |  |
| *Stutzerimonas stutzeri* |  |  |  |  |  |  |  |  |  |  |  |  |  |  |  |  |  |  |  |  |
| *Shewanella algae* |  |  |  |  |  |  |  |  |  |  |  |  |  |  |  |  |  |  |  |  |
| *Shewanella chilikensis* |  |  |  |  |  |  |  |  |  |  |  |  |  |  |  |  |  |  |  |  |
| *Shewanella hafniensis* |  |  |  |  |  |  |  |  |  |  |  |  |  |  |  |  |  |  |  |  |
| *Shewanella putrefaciens* |  |  |  |  |  |  |  |  |  |  |  |  | √ |  |  |  |  |  |  |  |
| *Shewanella xiamenensis* |  |  |  |  |  |  |  |  |  |  |  |  |  |  |  |  |  |  |  |  |
| *Staphylococcus epidermidis* |  |  |  |  |  |  |  |  |  |  |  |  |  |  |  |  |  |  |  |  |
| *Vibrio alginolyticus* |  |  |  |  |  |  |  |  |  |  |  |  |  |  |  |  |  |  |  |  |
| *Vibrio azureus* |  |  |  |  |  |  |  |  |  |  |  |  |  |  |  |  |  |  |  |  |
| *Vibrio fluvialis* |  |  |  |  |  |  | √ |  |  |  |  |  |  |  |  |  |  |  |  |  |
| *Vibrio neocaledonicus* |  |  |  |  |  |  |  |  |  |  |  |  |  | √ |  |  |  |  |  |  |
| *Vibrio parahaemolyticus* |  |  |  |  |  |  |  |  |  |  |  |  |  |  | √ |  |  |  |  |  |

**Supplementary Table 6** **Distribution of the isolated fungi in the hemolymph and muscle tissues of each WMS and Healthy crab**

|  | WMS 1 | | WMS 2 | | WMS 3 | | WMS 4 | | WMS 5 | | WMS 6 | | WMS 7 | | WMS 8 | | WMS 9 | | WMS 10 | |
| --- | --- | --- | --- | --- | --- | --- | --- | --- | --- | --- | --- | --- | --- | --- | --- | --- | --- | --- | --- | --- |
|  | Hem. | M | Hem. | M | Hem. | M | Hem. | M | Hem. | M | Hem. | M | Hem. | M | Hem. | M | Hem. | M | Hem. | M |
| *Cladosporium cycadicola* |  |  |  |  |  |  |  |  |  |  |  |  |  |  |  |  | √ |  |  |  |
| *Curvularia petersonii* |  |  |  |  |  |  | √ |  |  |  | √ |  |  |  |  |  |  |  |  |  |
| *Debaryomyces hansenii* |  |  |  |  |  |  |  |  |  |  | √ |  |  |  |  |  |  |  |  |  |
| *Hortaea werneckii* |  |  |  |  |  |  |  |  |  |  |  |  |  |  |  |  |  |  |  |  |
| *Lodderomyces elongisporus* |  | √ |  |  |  | √ |  |  |  | √ |  |  | √ |  |  |  |  |  | √ | √ |
| *Macrocybe gigantea* | √ |  |  |  |  |  | √ |  | √ |  |  |  |  |  | √ |  |  |  |  |  |
| *Meyerozyma guilliermondii* |  |  |  |  |  |  |  | √ |  |  |  | √ |  | √ |  |  |  |  |  |  |
| *Penicillium goetzii* |  |  |  |  | √ |  |  |  |  |  |  |  | √ |  | √ |  |  |  |  |  |
| *Penicillium rubens* |  |  |  | √ |  |  |  |  |  |  |  | √ |  |  | √ | √ |  | √ | √ |  |
| *Simplicillium sympodiophorum* |  |  | √ |  |  |  |  |  | √ |  |  |  |  |  |  |  |  |  |  |  |
| *Trichoderma effusum* | √ |  |  |  |  |  |  |  |  |  |  |  |  |  |  |  |  |  |  |  |
| *Trichoderma gamsii* |  |  |  |  |  |  |  |  |  |  |  |  |  |  |  |  |  |  |  |  |
| *Trichoderma saturnisporum* |  |  | √ |  | √ |  |  |  |  |  |  |  |  |  |  |  | √ |  |  |  |
| *Wickerhamomyces anomalus* |  |  |  |  |  |  |  |  |  |  |  |  |  |  |  |  |  |  |  |  |
|  | Healthy 1 | | Healthy 2 | | Healthy 3 | | Healthy 4 | | Healthy 5 | | Healthy 6 | | Healthy 7 | | Healthy 8 | | Healthy 9 | | Healthy 10 | |
|  | Hem. | M | Hem. | M | Hem. | M | Hem. | M | Hem. | M | Hem. | M | Hem. | M | Hem. | M | Hem. | M | Hem. | M |
| *Cladosporium cycadicola* |  |  |  | √ |  |  |  |  |  |  |  |  |  | √ |  |  |  | √ |  | √ |
| *Curvularia petersonii* |  |  |  |  |  |  | √ |  |  |  |  |  |  |  |  |  |  |  |  |  |
| *Debaryomyces hansenii* |  |  |  |  |  |  |  | √ |  | √ |  |  |  |  |  |  |  |  |  |  |
| *Hortaea werneckii* |  |  | √ |  |  |  |  |  |  |  | √ |  |  |  |  |  |  |  |  |  |
| *Lodderomyces elongisporus* |  |  |  |  |  |  |  |  |  |  |  |  |  |  |  |  |  |  |  |  |
| *Macrocybe gigantea* |  |  |  |  |  |  |  |  |  |  |  |  |  |  |  |  |  |  |  |  |
| *Meyerozyma guilliermondii* |  |  |  |  | √ |  |  |  | √ |  |  |  |  |  |  |  |  |  |  |  |
| *Penicillium goetzii* |  |  |  |  |  |  |  |  |  |  |  |  |  |  |  |  |  |  |  |  |
| *Penicillium rubens* |  |  |  |  |  |  |  |  |  |  | √ |  | √ |  |  |  |  |  |  |  |
| *Simplicillium sympodiophorum* |  |  |  |  |  |  |  |  |  |  |  |  |  |  | √ |  |  |  |  |  |
| *Trichoderma effusum* |  |  |  |  |  |  |  |  |  |  |  |  |  |  |  |  |  |  |  |  |
| *Trichoderma gamsii* |  | √ |  |  |  |  | √ |  |  |  |  |  |  |  |  |  |  |  |  |  |
| *Trichoderma saturnisporum* | √ |  |  |  |  |  |  |  |  |  |  |  |  |  |  |  |  |  |  |  |
| *Wickerhamomyces anomalus* |  |  |  |  |  |  |  |  |  |  |  |  | √ |  |  |  |  |  | √ |  |

**Supplementary Table 7** **Sample information for 13 WMS and 10 Healthy selected crabs**

|  | Hemolymph | Muscle |
| --- | --- | --- |
| Healthy1 | HH1 | HM1 |
| Healthy2 | HH2 | HM2 |
| Healthy3 | HH3 | HM3 |
| Healthy4 | HH4 | HM4 |
| Healthy5 | HH5 | HM5 |
| Healthy6 | HH6 | HM6 |
| Healthy7 | HH7 | HM7 |
| Healthy8 | HH8 | HM8 |
| Healthy9 | HH9 | HM9 |
| Healthy10 | HH10 | HM10 |
| Disease1 | DH1 | DM1 |
| Disease2 | DH2 | DM2 |
| Disease3 | DH3 | DM3 |
| Disease4 | DH4 | DM4 |
| Disease5 | DH5 | DM5 |
| Disease6 | DH6 | DM6 |
| Disease7 | DH7 | DM7 |
| Disease8 | DH8 | DM8 |
| Disease9 | DH9 | DM9 |
| Disease10 | DH10 | DM10 |
| Disease11 | DH11 | DM11 |
| Disease12 | DH12 | DM12 |
| Disease13 | DH13 | DM13 |

**Supplementary Table 8** **Sample information for the 16S rRNA and ITS amplicon sequencing in the hemolymph of WMS and Healthy crabs**

| Bacteria (16S rRNA sequencing) | | Fungi (ITS sequencing) | |
| --- | --- | --- | --- |
| HH1 | DH3 | HH1 | DH3 |
| HH2 | DH4 | HH2 | DH5 |
| HH4 | DH5 | HH4 | DH7 |
| HH6 | DH6 | HH7 | DH8 |
| HH7 | DH7 | HH9 | DH10 |
| HH8 | DH8 | HH10 | DH11 |
| HH9 | DH9 |  | DH13 |
| HH10 | DH10 |  |  |
|  | DH11 |  |  |
|  | DH13 |  |  |
| n = 8 | n = 10 | n = 6 | n = 7 |

**Supplementary Table 9** **Primer pairs of RT-qPCR for six bacteria which cause WMS in twice regression infection experiments**

| Table S9 The primer pairs of RT-qPCR for the six bacteria which could cause WMD in twice regression infection experiments | | | | | |
| --- | --- | --- | --- | --- | --- |
|  | Spcies | Forward primer | Reverse primer | Tm-F (℃) | Tm-R (℃) |
| Tthe first amplification | *Shewanella* *chilikensis* | ACTGGGCAACAGATACGCAA | AGTGCGTCAGGGAGACAATG | 59.96 | 60.04 |
|  | *Shewanella* *putrefaciens* | TGTCAATGCGGGCAAACAAG | TCGACGGCATCGGTATTGAG | 59.97 | 59.97 |
|  | *Shewanella* *xiamenensis* | GAAATCCAAGCTGCGCTACG | CAAATAATCATGGCGGCGCA | 59.97 | 59.97 |
|  | *Vibrio* *alginolyticus* | TGCGCCTGTCACATTATGGT | CGCTTTGCGATAGAACGTGG | 60.04 | 59.97 |
|  | *Vibrio* *fluvialis* | GTACGAAATTCGCGACCGTG | CGCCAAAACCCAGCATATCG | 59.98 | 59.97 |
|  | *Vibrio* *parahaemolyticus* | CACAACACAACTGGCGAAGG | TACGCGGTAAGGATCGAACG | 59.97 | 59.97 |
| The second amplification | *Shewanella* *chilikensis* | AATGCGAGATTTGAGCCGTG | AACCCTGAAACGCGATGGTT | 59.27 | 60.54 |
|  | *Shewanella* *putrefaciens* | AAGGTGAATCCCTGTGGAGC | CACCGGCTCTGCTAACATCA | 59.67 | 60.11 |
|  | *Shewanella* *xiamenensis* | TAAATGGTTGCGCAATGCCG | GGTTGCGCTGGCAATAAGTT | 60.46 | 59.76 |
|  | *Vibrio* *alginolyticus* | TGGTTCATCAGTTGCTGGCT | CGTCTTCCGTGAATGGGTCA | 59.89 | 60.04 |
|  | *Vibrio* *fluvialis* | GGCATGAACGTGCTGGAATC | AGACCTGGACCACGGTAAGA | 59.9 | 59.89 |
|  | *Vibrio* *parahaemolyticus* | ACACACTCCACTCAAGCGTT | TAACGGTCTGCGGCCATAAA | 59.82 | 59.75 |
