# Supplementary material for: Integration of microbiome and Koch’s postulates to reveal multiple bacterial pathogens of whitish muscle syndrome in mud crab, Scylla paramamosain
Source: Microbiome. 2023 Jul 20;11:155. doi: 10.1186/s40168-023-01570-6 (PMC10357871; doi:10.1186/s40168-023-01570-6)
Supplement: Supplementary file 3 — Additional file 2: Fig. S1. A large number of bacteria (the red arrow points to the blue dot) but no parasites were present in the muscle of WMS crabs using the brightfield, fluorescence & FISH digital pathology scanner (Lecia, Versa 8). Fig. S2. Bacterial and fungal community of WMS. a Rarefaction and Shannon curve. b Bacterial and fungal community structure of WMS and Healthy crabs based on Bray-Curtis distance. c Bacterial and fungal indicator genera in the hemolymph for WMD. *: P < 0.05, ***: P <0.001. Fig. S3. Phylogenetic tree of all 23 bacterial species isolated. Fig. S4. Sequence chart of WMS in each infection group during the second regression infection experiment. Fig. S5. Phylogenetic tree of S. chilikensis, S. putrefaciens, S. xiamenensis, V. alginolyticus, V. fluvialis, and V. parahaemolyticus re-isolated which cause WMS in twice regression infection experiments. [file 40168_2023_1570_MOESM2_ESM.docx]

**Supplementary Figures**

**
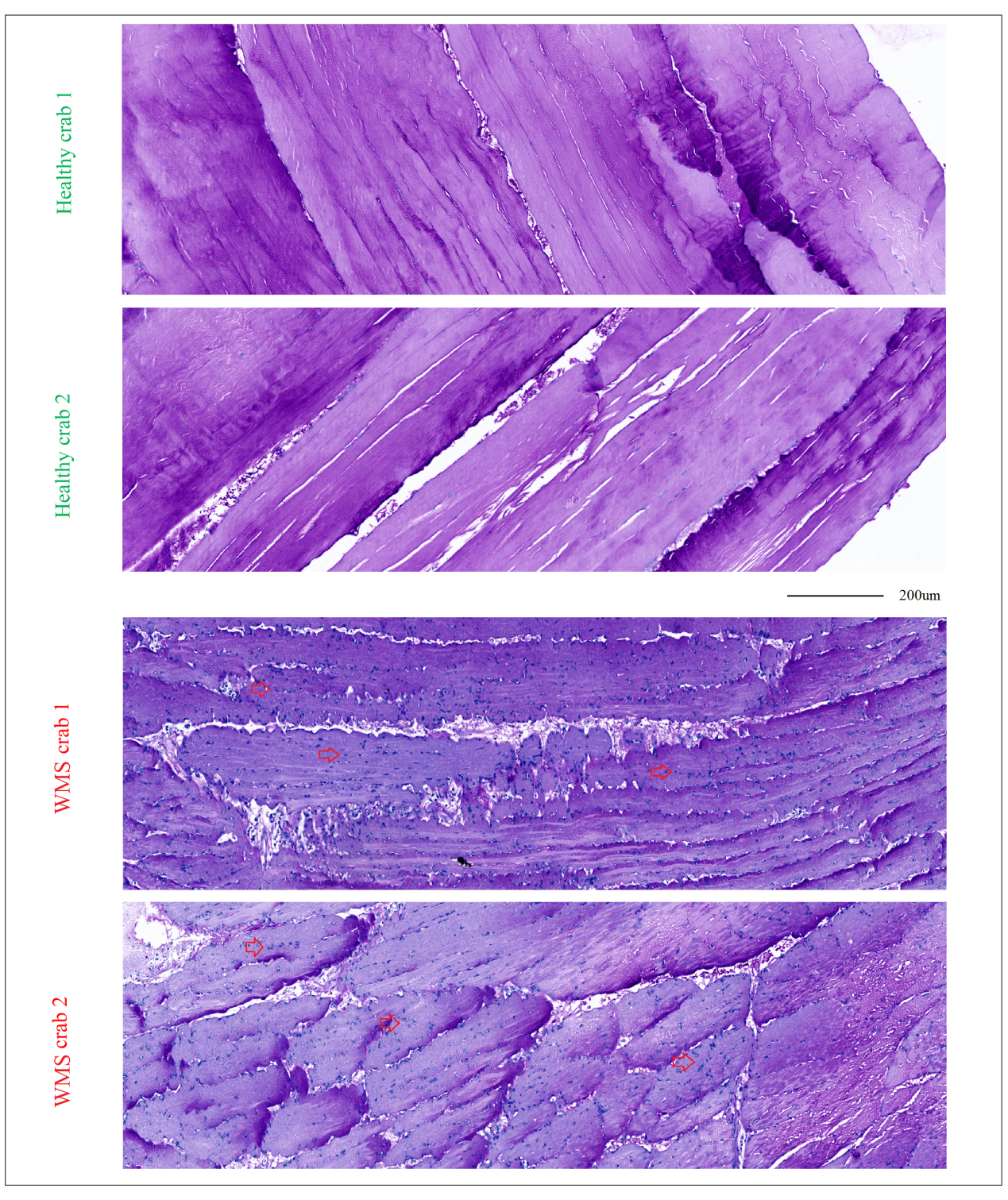
**

**Supplementary Figure 1** **A large number of bacteria (the red arrow points to the blue dot) but no parasites were present in the muscle of WMS crabs using the brightfield, fluorescence & FISH digital pathology scanner (Lecia, Versa 8).**

**
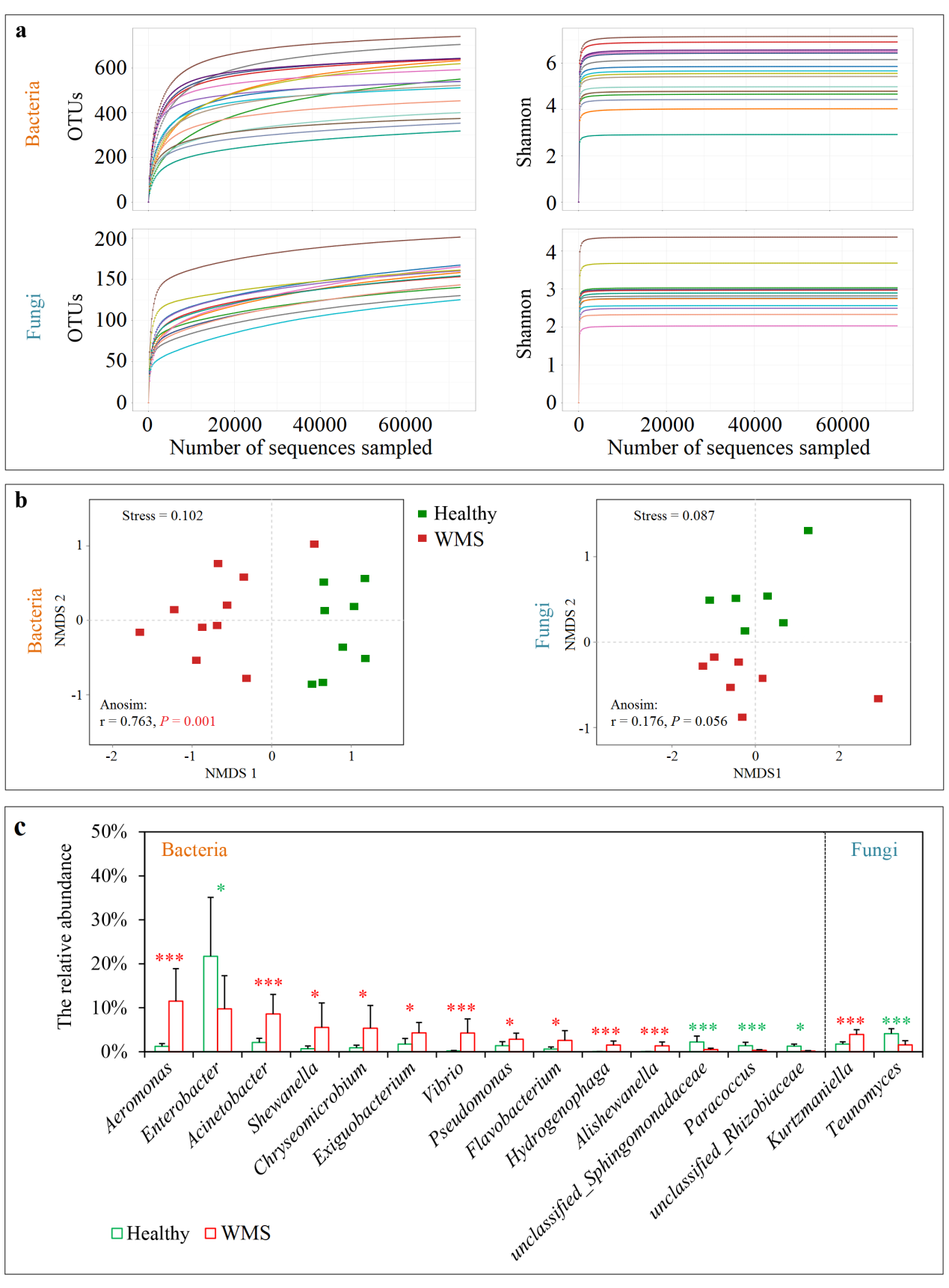
Supplementary Figure 2 Bacterial and fungal community of WMS.** **a** Rarefaction and Shannon curve. **b** Bacterial and fungal community structure of WMS and Healthy crabs based on Bray-Curtis distance. **c** Bacterial and fungal indicator genera in the hemolymph for WMD. *: *P* < 0.05, ***: *P* < 0.001

**
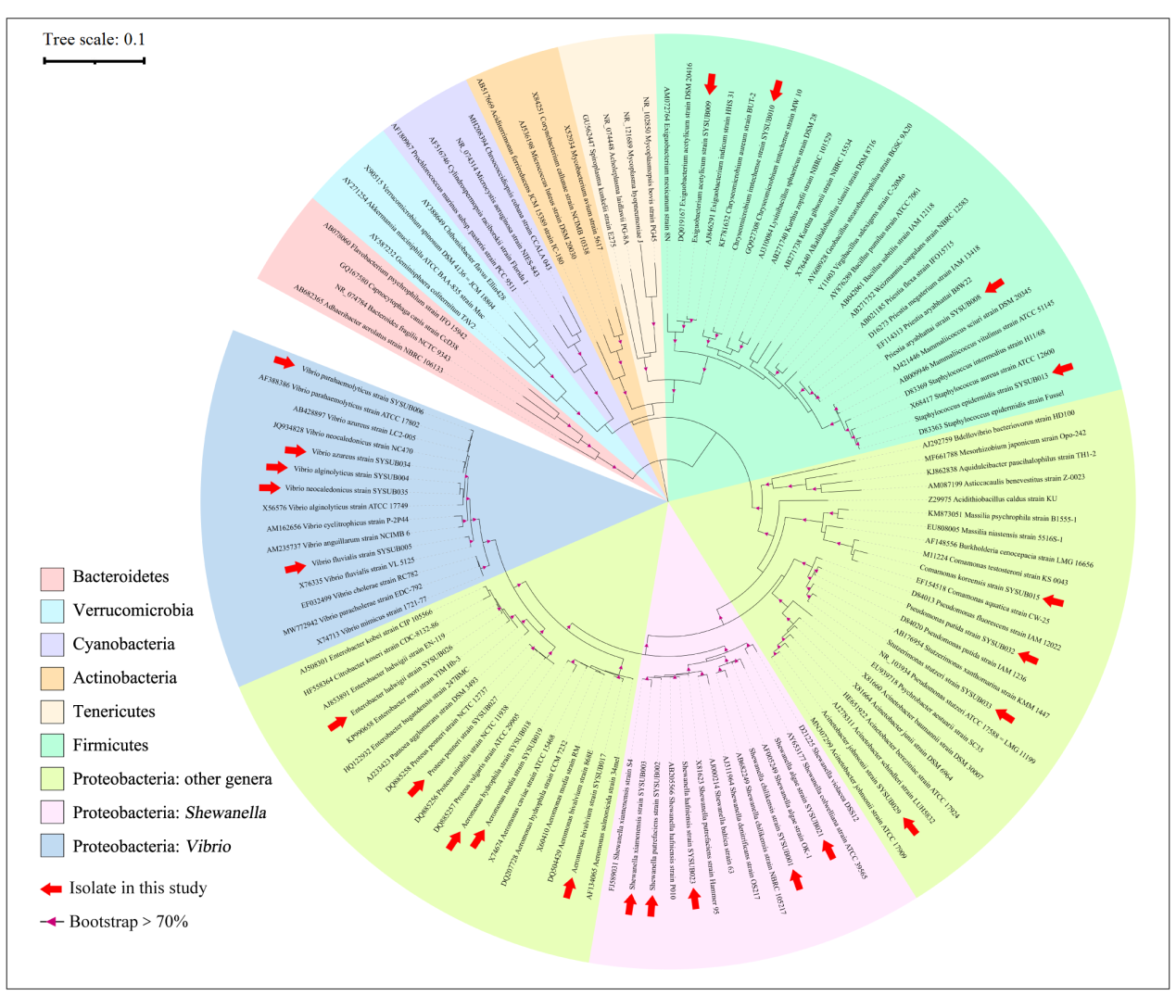
**

**Supplementary Figure 3** **Phylogenetic tree of all 23 bacterial species isolated.**

**
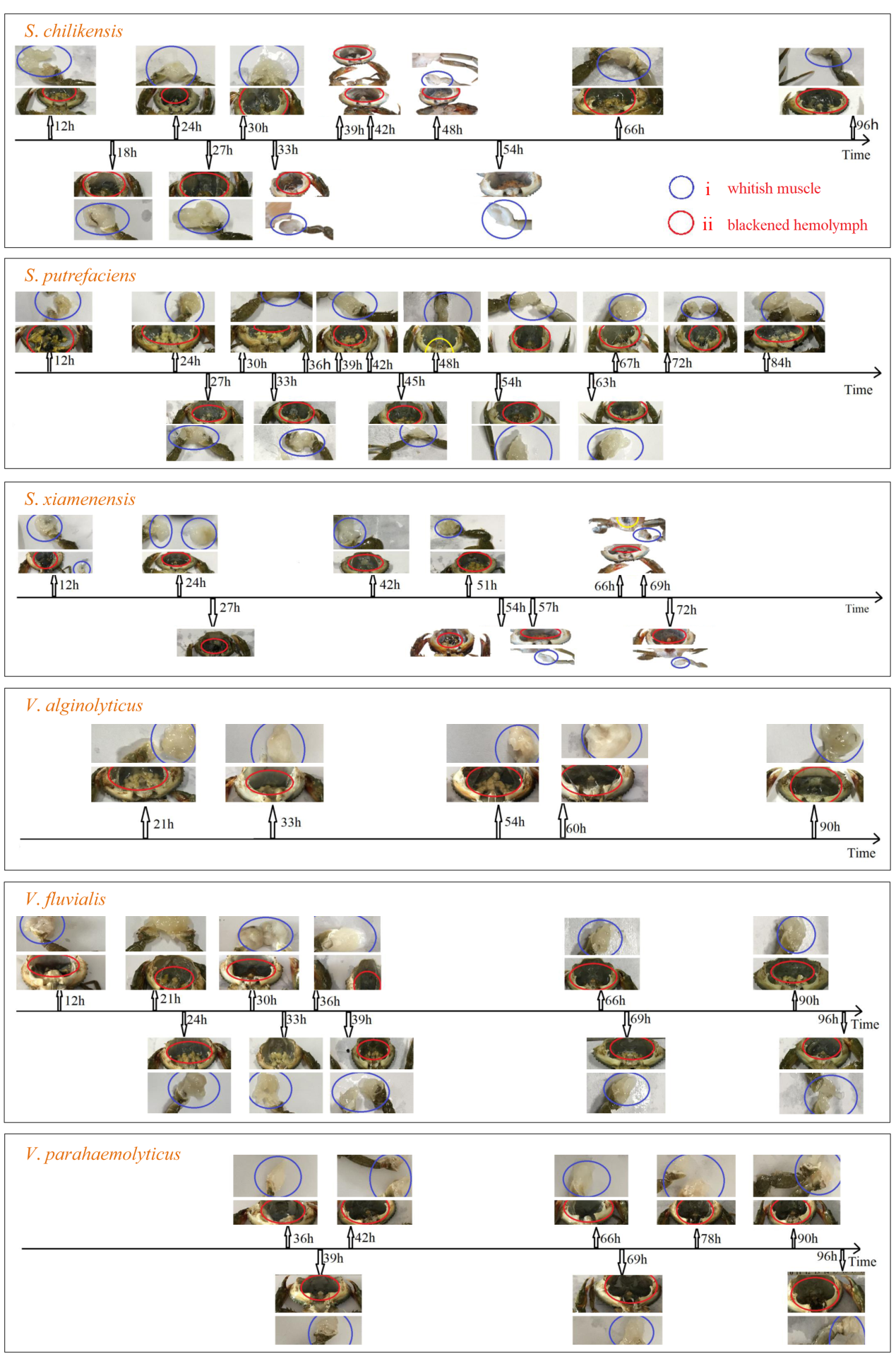
**

**Supplementary Figure 4 Sequence chart of WMS in each infection group during the second regression infection experiment.**

**
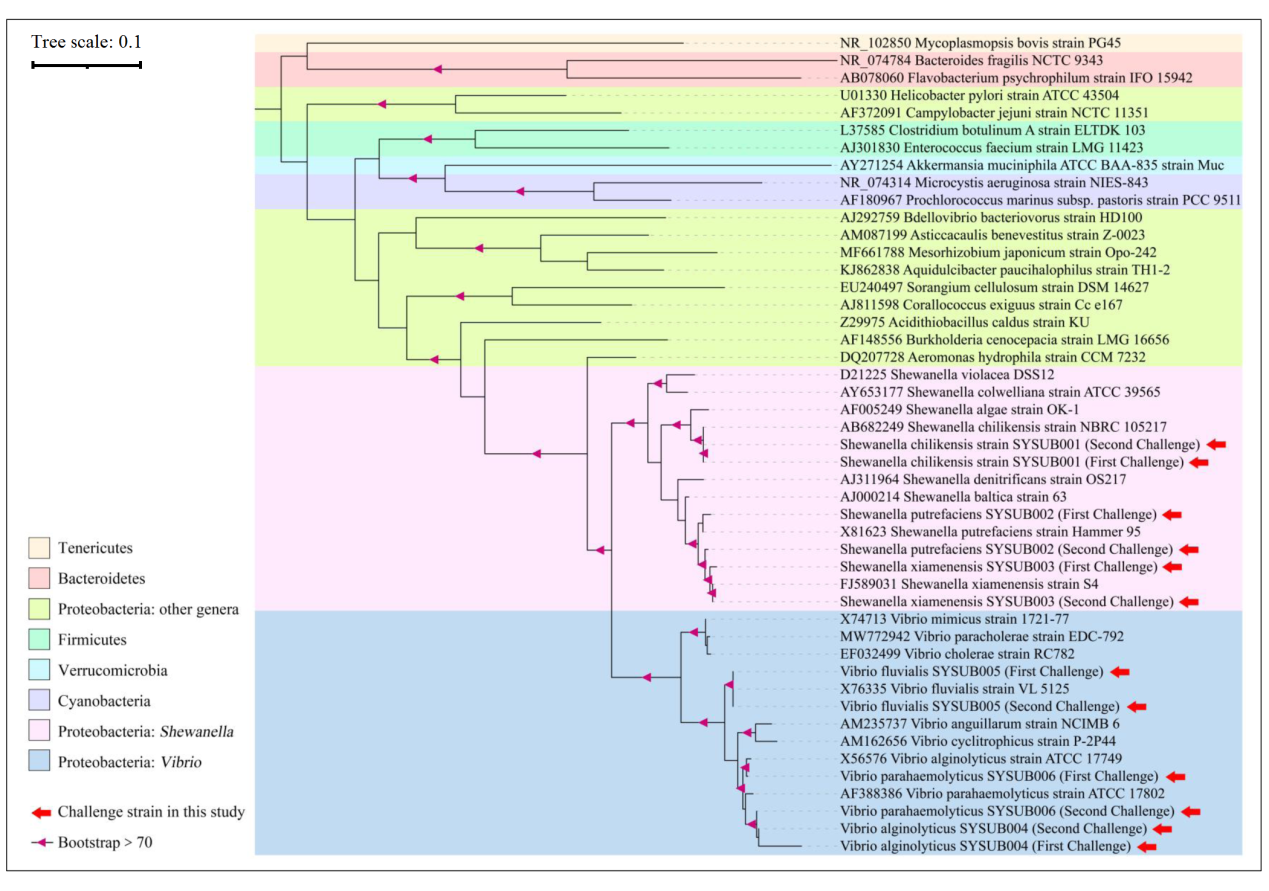
**

**Supplementary Figure 5 Phylogenetic tree of *S*. *chilikensis*, *S*. *putrefaciens*, *S*. *xiamenensis*, *V*. *alginolyticus*, *V*. *fluvialis*, and *V*. *parahaemolyticus* re-isolated which cause WMS in twice regression infection experiments.**
